# AI-designed prion-capping proteins provide evidence that prion fibril ends are replication-competent surfaces that contribute to prion seeding activity and infectivity

**DOI:** 10.1128/mbio.00831-26

**Published:** 2026-08-17

**Authors:** Jessy A. Slota, Matthew Kirby, Aidan Van Den Driessche, Dominic M. S. Kielich, Lise Lamoureux, Daniel R. Beniac, Christine Layne, Melissa Poirier, Jörg Stetefeld, Stephanie A. Booth, Ben A. Bailey-Elkin

**Affiliations:** 1 High Consequence Pathogens Division, National Microbiology Laboratory, Public Health Agency of Canada 85072 , Winnipeg, Manitoba, Canada; 2 Department of Chemistry, Faculty of Science, University of Manitoba 428123, Winnipeg, Manitoba, Canada; 3 Scientific Core Services Division, National Microbiology Laboratory, Public Health Agency of Canada 85072 , Winnipeg, Manitoba, Canada; 4 Department of Medical Microbiology and Infectious Diseases, Faculty of Health Sciences, University of Manitoba 574854, Winnipeg, Manitoba, Canada; Duke University School of Medicine, Durham, North Carolina, USA

**Keywords:** prion, Creutzfeldt-Jakob disease, RFdiffusion, amyloid

## Abstract

End-elongation of amyloid fibrils is a prevailing theory to explain prion replication, but direct experimental evidence for this phenomenon is limited by the lack of research tools. To serve as molecular probes for prion fibril termini, here, we designed protein binders against high-resolution structures of infectious prion fibrils using a diffusion-based design model. By generating protein scaffolds around short β-strand segments from terminal prion rungs, we designed β-hairpin-interfacing proteins that cap prion fibrils, which we termed PRICAPs. We validated that PRICAPs exhibit binding to prion fibril termini and confirmed the role of prion fibril ends in replication by demonstrating that PRICAPs inhibit prion seeding activity and attenuate prion replication in organotypic cerebellar slice cultures. Collectively, these findings describe a class of prion-capping protein that can be used to probe prion fibril termini and verify that these surfaces contribute to prion replication and infectivity.

**IMPORTANCE:**

Prions replicate by templating the misfolding of native proteins; however, direct evidence identifying the precise sites of replication has remained limited. Here, we address this gap by developing a new class of rationally designed protein tools that specifically bind and cap the ends of infectious prion fibrils. Using these probes, we demonstrate that fibril termini are replication-competent surfaces required for prion seeding and propagation. By inhibiting these sites, our designed proteins markedly reduce prion replication in a disease-relevant *ex vivo* system, providing functional validation of the end-elongation model. Beyond resolving a fundamental question in prion biology, this work establishes a generalizable strategy for targeting amyloid fibril ends with high specificity. These findings have broad implications for understanding protein aggregation in neurodegenerative diseases and open new avenues for the development of therapeutics that selectively disrupt pathogenic amyloid propagation.

## INTRODUCTION

Prion diseases are rare transmissible neurodegenerative disorders caused by the accumulation of misfolded prion protein (PrP^Sc^) in the brain, which progress rapidly and are universally fatal (1). PrP^Sc^ is thought to arise from the conformational conversion of α-helical cellular prion proteins (PrP^C^) into β-sheet-rich amyloid fibrils (2). Recently solved cryogenic electron microscopy structures reveal that infectious prion fibrils adopt a parallel in-register β-sheet (PIRIBS) arrangement, where rungs derived from individual PrP monomers stack as continuous β-sheets to form fibrils (3–8). Fibril termini are assumed to be replication-competent surfaces where additional PrP^C^ monomers are bound and converted into the pathological conformer, but this has yet to be demonstrated experimentally.

Mechanistic studies of prion replication are hindered by a lack of binders that exhibit specificity toward disease-associated prion conformations, which could also be applied to prion enrichment and detection, or inform therapeutic strategies. Most antibodies recognize PrP^C^ or linear epitopes, like 3F4 (9), the SAF series (10), the POM monoclonals (11), and the ICSM antibodies (12). These antibodies lack specificity for PrP^Sc^, and some may be neurotoxic when binding PrP^C^ (13). A few PrP^Sc^-specific antibodies were also reported (14–18), but some can cross-react with other amyloids (e.g., amyloid-β) and remain challenging to generate.

Binder design has greatly benefited from recent advances in deep learning models, which allow *in silico* design of complete protein domains. RFdiffusion (19) is one such deep-learning framework that has shown success in generating amyloidogenic peptide traps (20) and binders of intrinsically disordered peptides (21), but it has not yet been applied to amyloid fibrils. Therefore, RFdiffusion-mediated targeting of binders to existing prion fibril structures might yield PrP^Sc^-specific binding proteins that engage amyloid-specific motifs at fibril termini, potentially hindering fibril growth.

To probe prion fibril termini, here, we used RFdiffusion to design protein scaffolds around short β-strand segments from terminal prion fibril rungs, yielding small β-hairpin proteins that cap fibril ends mainly through backbone hydrogen-bonding networks. Design campaigns generated a library of 28,500 protein binders targeting terminal surfaces at N-terminal and C-terminal lobes of the existing prion PIRIBS structures (3–8). For experimental validation, we expressed and characterized 30 prion capping proteins, named PRICAP 1–30. Dot blot assays and electron microscopy with gold nanoparticle (GNP) labeling verified the fibril end-specific binding mechanisms of PRICAPs, while real-time quaking-induced conversion (RT-QuIC) assays confirmed their ability to inhibit prion seeding activity. Importantly, PRICAPs attenuated prion replication kinetics in organotypic cerebellar slice cultures, verifying their functionality *ex vivo*. Together, these findings demonstrate that structures of prion fibril termini can be effectively targeted by generative models to design fibril-capping proteins that impair prion replication. More broadly, by linking prion fibril termini with seeding activity and infectivity, our study provides evidence of the end-elongation model for prion fibril growth.

## RESULTS

### Structure-guided design of small proteins to cap prion fibril ends

**Fig 1:**
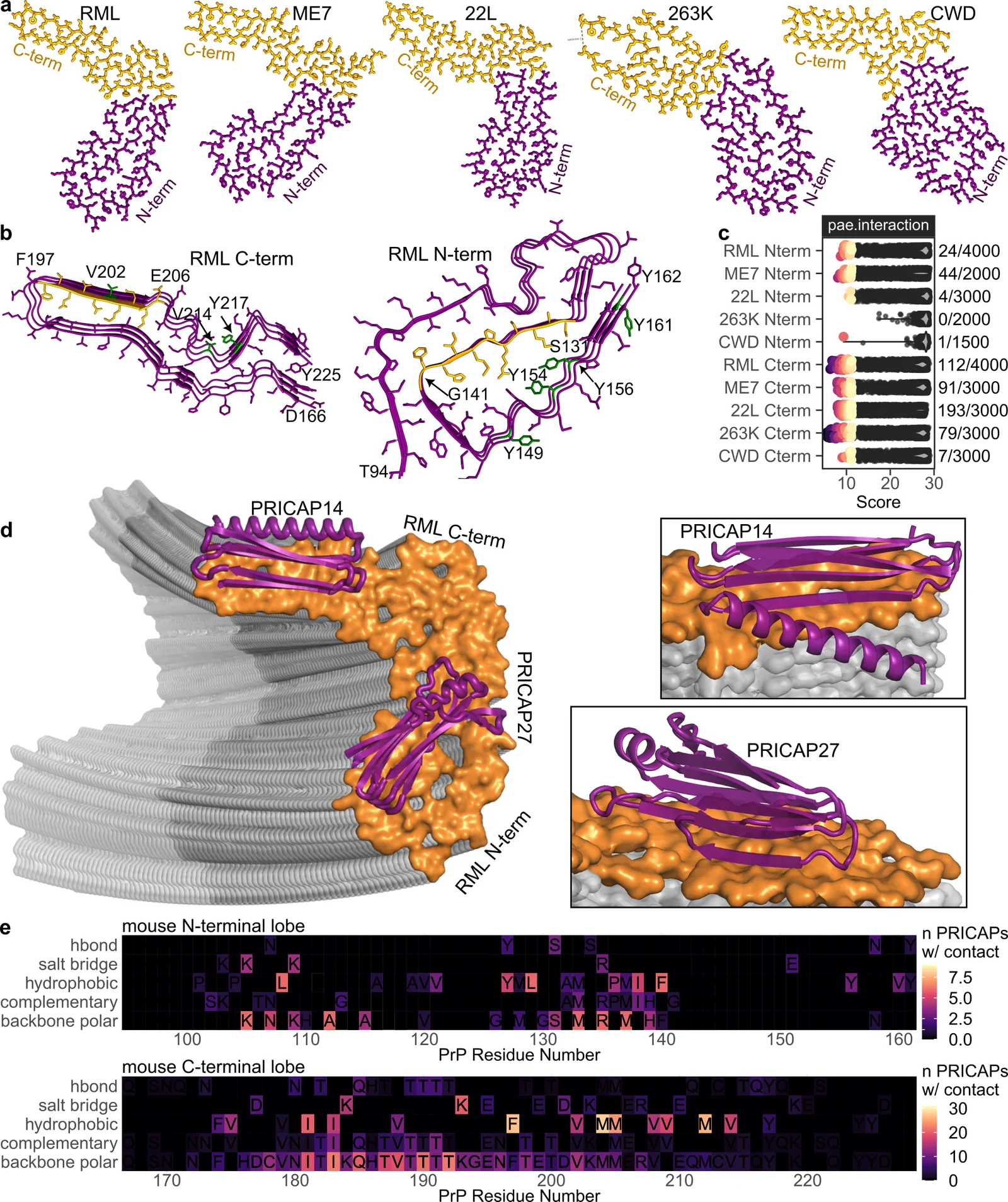
RFdiffusion-mediated design of proteins that cap prion fibril ends. (**a**) Targets comprised five prion structures (RML, ME7, 22L, 263K, and CWD) split into N- and C-terminal 3-rung lobes. (**b**) RML’s N- and C-terminal lobes annotated with inpainted regions (yellow) and hotspot residues (green). All annotated fibril lobes are shown in Fig. S1. (**c**) RFdiffusion campaigns designed 1,500–4,000 binders per target that were ranked using AlphaFold2 pae.interaction scores, with scores below 11.4 considered putative prion-capping proteins (PRICAPs). Annotated are the number of structures with PAE <11.4/total number of designs. Additional AlphaFold2 metrics are provided in Fig. S2. (**d**) Model of representative PRICAPs bound to their respective C-terminal (PRICAP14) and N-terminal (PRICAP27) target lobes on an RML fibril, with additional structures in Figs. S3 and S4. (**e**) Heatmap showing frequent mouse PrP-PRICAP residue contacts across all *in silico*-validated PRICAPs designed against RML, ME7, or 22L. Shown are the number of PRICAPs that make contacts with each PrP residue through hydrogen bonding (hbond), salt bridges, hydrophobic stacking, polar backbone interactions, and whether PRICAP-PrP contacting residues match (complementary).

To interfere with prion fibril elongation, we sought to design prion-capping proteins, or PRICAPs. We therefore explored the use of RFdiffusion with published prion PIRIBS structures as targets, including RML (3, 4), ME7 (5), and 22L (6) mouse-adapted scrapie, 263K hamster-adapted scrapie (7), and natural white-tailed deer chronic wasting disease (8). Targets comprised three PrP rungs of either the N-terminal or C-terminal fibril lobe (Fig. 1a). As recommended, we used ProteinMPNN (22) to thread sequences onto diffused structures followed by AlphaFold2 initial guess (23) for *in silico* binding validation.

We reasoned that effective PRICAPs would form β-sheets that interface with terminal fibril rungs, and hence, we used RFdiffusion’s “Complex_Beta” model for binder design. To control the location of the binding interface, our diffusion strategy also employed structure inpainting, which preserves the backbone conformation of a designated region on the protein target but replaces the amino acid sequence. Specifically, we inpainted a short β-strand from the top or bottom prion rung, flanked it with newly diffused structure, and selected binding hotspots from hydrophobic residues on the adjacent fibril rung. To illustrate our design strategy, Fig. 1b shows RML’s N- and C-terminal lobes labeled with inpainted and hotspot residues, and Fig. S1 shows similar annotated structures for all 10 targets.

We produced a library of 28,500 putative PRICAPs through campaigns designing 1,500–4,000 binders against each of the 10 target structures. AlphaFold2 metrics were used to assess *in silico* success rates (Fig. 1c; Fig. S2a) across targets. C-terminal lobes outperformed their N-terminal counterparts. Filtering based on AlphaFold2 metrics identified 159 PRICAP candidates for further consideration.

Analysis of predicted PRICAP structures revealed class-defining characteristics (Fig. 1d and e; Fig. S2b through d and S3). Each PRICAP comprises several β-strands, usually arranged as two β-sheets that interface to form a β-hairpin, which mimics the structure of the targeted fibril lobe. These PRICAP β-sheets interface with terminal PrP PIRIBS rungs through backbone hydrogen-bonding networks, usually spanning mouse PrP residues K105–F140 in N-terminal lobe binders and H176–M205 in C-terminal lobe binders, sometimes with complementary PRICAP-PrP-interfacing residues. Hydrophobic interactions were also common among PRICAP-PrP contacts, while salt bridges and side-chain hydrogen bonding were less common. Predicted structures of representative PRICAPs bound to their target prion lobe are shown in Fig. 1d; Fig. S4 and S5, and all metrics on the 159 PRICAP candidates are provided in File S1.

### Production and *in vitro* characterization of prion-capping proteins

**Fig 2:**
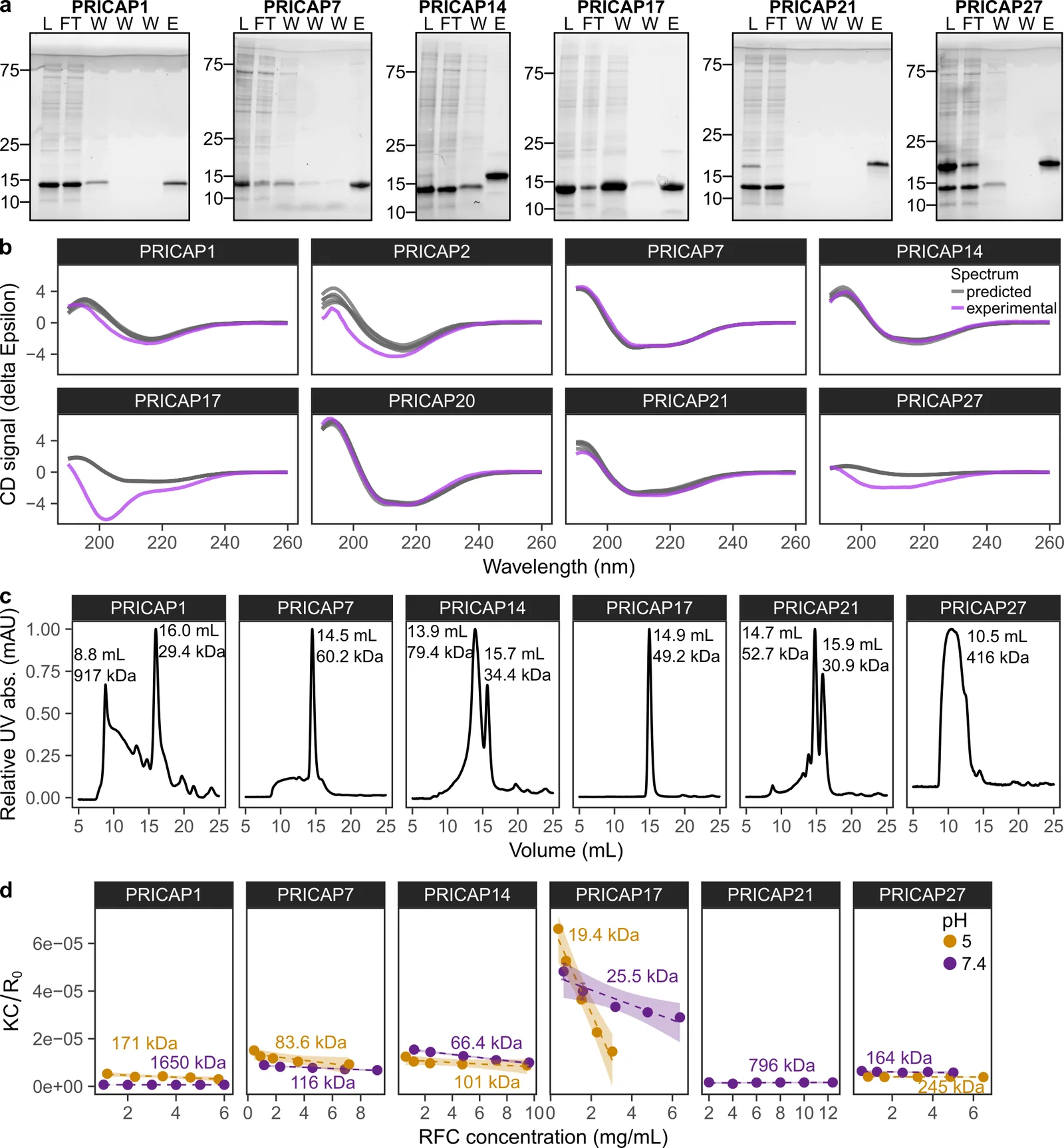
Production and characterization of PRICAPs. (**a**) Representative SDS-PAGE analyses of *E. coli* lysate (L), flow-through (FT), wash (W), and elution (E) fractions from purifications of twin-strep-tagged PRICAPs using Streptactin-XT resin. (**b**) CD spectroscopy of PRICAPs, comparing experimental with predicted spectra of the top five AlphaFold3 models. (**c**) Representative size exclusion chromatography (SEC) profiles of purified PRICAPs run on a Superdex 200 Increase 10/300 GL in 50 mM Tris, pH 7.5, 150 mM NaCl. (**d**) Representative Debye plots annotated with molecular weights of PRICAPs in 50 mM Tris, pH 7.5, 150 mM NaCl, or 20 mM NaAc, pH 5.0.

Ultimately, we chose 30 putative prion-capping proteins for production and characterization, naming them PRICAP1 through PRICAP30. PRICAPs with C-terminal TwinStrep tags were produced in *Escherichia coli* and purified using Streptactin XT resin. We noted that inclusion bodies often formed during pilot induction experiments (Fig. S6), which sometimes limited yields of soluble protein. Consequently, soluble protein yield was considered when selecting PRICAPs for analysis in downstream assays. Figure 2a shows SDS-PAGE analyses of fractions collected during purification of representative PRICAPs.

To verify whether PRICAPs folded into their predicted structure, we collected circular dichroism spectra (Fig. 2b). We observed good agreement between experimental and predicted CD spectra for PRICAPs 7, 14, 20, and 21, suggesting that they were folded as expected. Conversely, PRICAPs 17 and 27 deviated from their predicted CD spectra, indicating inaccuracies in their predicted structures.

We also characterized PRICAPs using size exclusion chromatography (Fig. 2c) and static light scattering (Fig. 2d) of purified PRICAPs. These size analyses revealed that PRICAPs prominently formed oligomeric complexes on the scale of dimers to >100-mers. This oligomerization might be explained by the β-sheet-rich conformations adopted by PRICAPs.

### Dot blot assays identify PRICAPs that preferentially bind fibrillar over monomeric PrP

**Fig 3:**
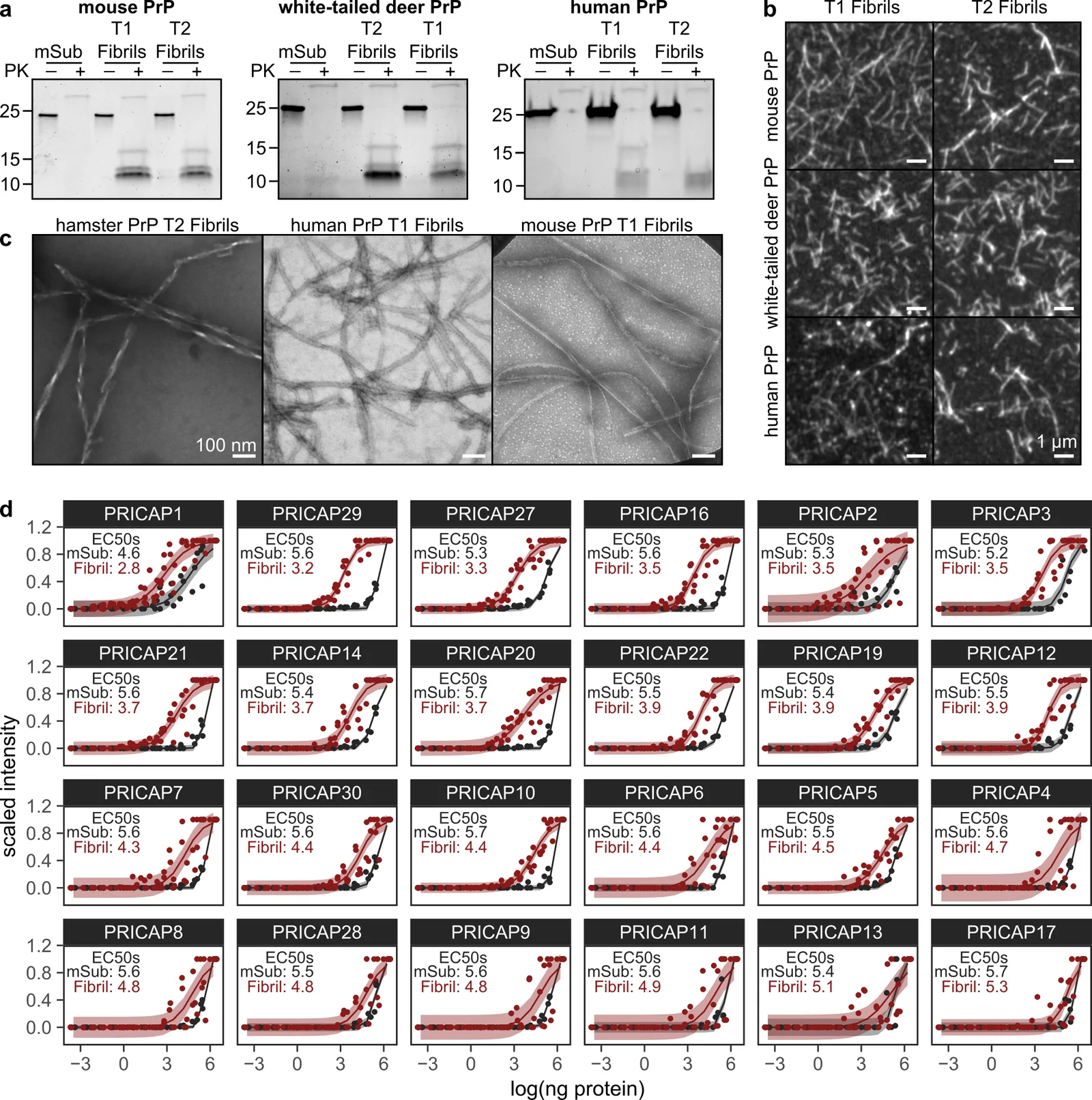
Dot blots demonstrate binding affinity and specificity of PRICAPs against synthetic prion fibrils. Prion fibril preparations, made from hamster, mouse, white-tailed deer, and human monomeric PrP substrates (mSub), were validated via (**a**) electrophoretic profiles without (−) and following proteinase K digestion (+), (**b**) confocal fluorescence microscopy following Thioflavin T (ThT) staining, and (**c**) negative stain electron microscopy. T1 and T2 fibrils were made with and without zirconium beads, respectively. To assess PRICAP-fibril binding, monomeric (mSub) and fibrillar PrP preparations were spotted onto nitrocellulose membranes and probed with purified PRICAPs diluted to 850 nM. (**d**) EC50 measurements of binding affinity were compared between monomeric and fibrillar PrP by fitting scaled dot blot intensity measurements against log(ng protein) using a four-parameter EC50 equation. Dot blot images are provided in Fig. S8.

To serve as *in vitro* targets to assess PRICAP binding, we produced synthetic recombinant prion fibrils through agitation under partial denaturing conditions in the absence of any polyanionic co-factors, which were validated using proteinase K digestion (Fig. 3a), confocal microscopy (Fig. 3b), and negative stain transmission electron microscopy (Fig. 3c). While these fibril preparations may not fully represent infectious brain-derived prions, they were produced consistently, remained soluble and stable at pH 5, and were of high purity.

To explore whether PRICAPs are capable of binding recombinant prion fibrils, we turned to dot blot assays using PRICAPs as probes against monomeric and fibrillar PrP. Preliminary dot blots were probed using crude PRICAP-containing bacterial lysates, which provided evidence that all 30 PRICAPs bound fibrillar PrP (Fig. S7). Therefore, to more robustly compare PRICAP binding between monomeric and fibrillar PrP, dot blots with more extensive PrP targets (3 PrP monomer + 5 PrP fibril targets × 15 dilutions) were probed using purified PRICAPs (Fig. S8). We arbitrarily included twin-strep-tagged Nrxn1 as a negative control to account for binding from the purification tag, although there is evidence that Nrxn1 can bind α-synuclein fibrils and amyloid-β oligos (24, 25). Of the 30 candidates, 24 PRICAPs were successfully purified and tested in this dot blot assay. To compare affinities, EC50 values were calculated via densitometry and curve fitting (Fig. 3d). As expected, all 24 PRICAPs showed stronger affinity to PrP fibrils than monomeric PrP. No signal was detected with Nrxn1 (Fig. S8), illustrating the relative strength of PRICAP-fibril binding.

**Fig 4:**
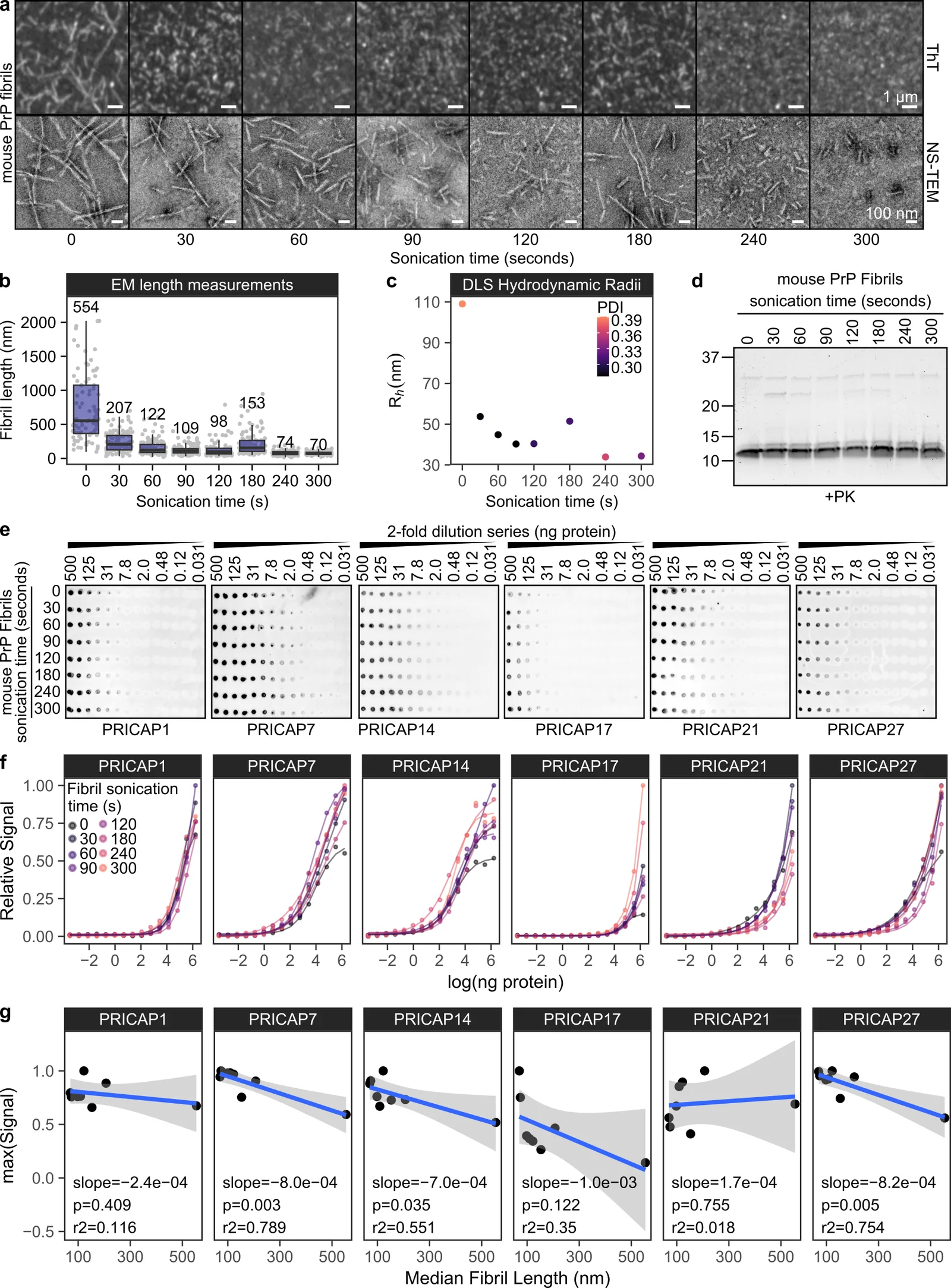
Relationship between recombinant prion fibril length and binding to PRICAPs. (**a**) Mouse PrP fibrils were sonicated for 0 to 300 s and visualized with confocal fluorescence microscopy after staining with ThT and NS-TEM. (**b**) Fibril lengths measured from NS-TEM images (annotated with median length, boxplots show median [center line], 25th and 75th percentiles [upper and lower hinges], and 1.5*IQR [whiskers]) and (**c**) hydrodynamic radii (Rh) from static light scattering are both inversely related to sonication time. (**d**) Proteinase K-resistant fragments in SDS-PAGE were unaffected by sonication. (**e–g**) Dot blots demonstrate enhanced binding of PRICAP7, PRICAP14, and PRICAP27 to fibrils of shorter length. Shown are (**e**) dot blot images, (**f**) signal curves, and (**g**) linear regression analysis of maximum dot blot signal intensity against median fibril length from NS-TEM images.

Interestingly, monomeric PrP often produced higher maximum signal intensities than fibrillar PrP despite the lower affinity for PRICAPs, which likely reflects the limited number of binding sites at fibril ends (one per single-stranded fibril) and consequently low stoichiometry of PRICAP-fibril binding. Therefore, we reasoned that fibrils of shorter length would have more binding sites and produce stronger signal in dot blot assays. Fibril preparations of different length distributions were made by sonicating recombinant mouse PrP fibrils for 0–300 s (Fig. 4a). Negative-stain transmission electron microscopy (TEM) confirmed a reduction in average fibril length from 746 to 75 nm (Fig. 4b), and dynamic light scattering (DLS) showed reduced hydrodynamic radii following sonication (Fig. 4c). Unaltered proteinase K resistance profiles confirmed that sonicated fibrils retained their amyloid characteristics (Fig. 4d). Dot blot assays demonstrated an inverse relationship between fibril length and signal intensity for PRICAP7, PRICAP14, and PRICAP27 (Fig. 4e through g). PRICAP21 was a notable outlier that exhibited reduced signal intensity with highly sonicated fibrils, perhaps because it formed very large oligomers. It is also possible that the sonication treatment could cause partial aggregation of the PrP filaments and occlusion of binding sites, which might mask the relationship between fibril length and binding affinity. Nevertheless, these findings are consistent with some PRICAPs binding to fibril ends.

### Gold nanoparticle labeling identifies PRICAPs bound to recombinant prion fibril ends

**Fig 5:**
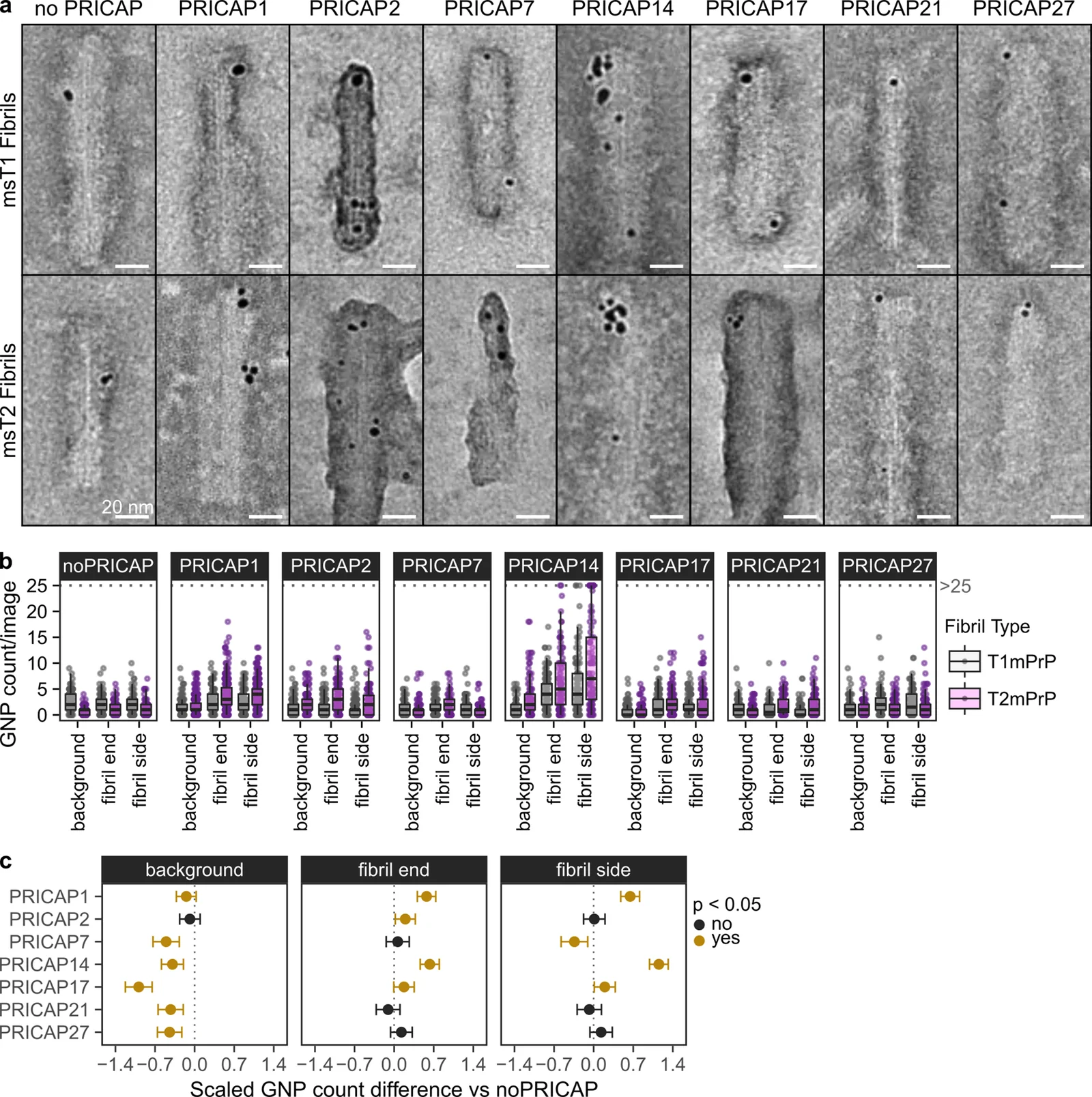
Electron microscopy with gold nanoparticle labeling identifies PRICAPs bound to recombinant prion fibril ends. (**a**) Representative negative-stain TEM images of sonicated recombinant mouse prion fibrils (type 1 and type 2) treated with PRICAPs (or no PRICAP) and then stained with streptactinXT-GNP conjugates. (**b**) Raw counts per image of GNPs associated with background, fibril sides, and fibril ends. Boxplots show median (center line), 25th and 75th percentiles (upper and lower hinges), and 1.5*IQR (whiskers). (**c**) Distributional shifts of background-, side-, and end-associated GNPs versus no PRICAP control estimated via a Poisson regression model. Shown are mean per-image GNP count differences with 95% confidence intervals.

To visualize whether PRICAPs bind fibrils via the predicted end-capping mechanism, we used GNP labeling to locate fibril-bound PRICAPs by TEM imaging. Recombinant prion fibrils were incubated with PRICAPs, deposited onto electron microscopy grids, stained with streptactin-GNP conjugate, and then visualized using negative-stain TEM (Fig. 5a; Fig. S9). Each GNP was classified as associated with fibril ends, fibril sides, or background, and per-image count distributions were compared between treatment groups using Poisson regression.

In preliminary experiments with human and white-tailed deer PrP fibrils, PRICAP treatments resulted in increased proportions of end-associated GNPs relative to untreated controls (Fig. S9). However, some individual GNPs were difficult to classify due to fibril clustering, dense GNP decoration, and image resolution. Non-specifically associated GNPs were also present in the negative control grids made without PRICAP, consistent with previous reports of GNPs binding directly to PrP (26, 27). Therefore, for more robust quantification, we generated a larger GNP data set using sonicated mouse PrP fibrils, which have more fibril ends available for binding. Across these experiments, PRICAP-treated fibril grids often presented with lower proportions of background-associated GNPs and higher frequencies of fibril-end-associated GNPs relative to controls (Fig. 5b and c). Fibril-end and side-associated GNPs were especially abundant in PRICAP14-treated grids, consistent with binding.

Thus, engagement of PRICAPs with prion fibril termini is supported by the distributional shifts in GNP localization toward enrichment at fibril ends seen across several PRICAPs and multiple fibril types.

### PRICAPs impede prion-seeding activity in RT-QuIC assays

**Fig 6:**
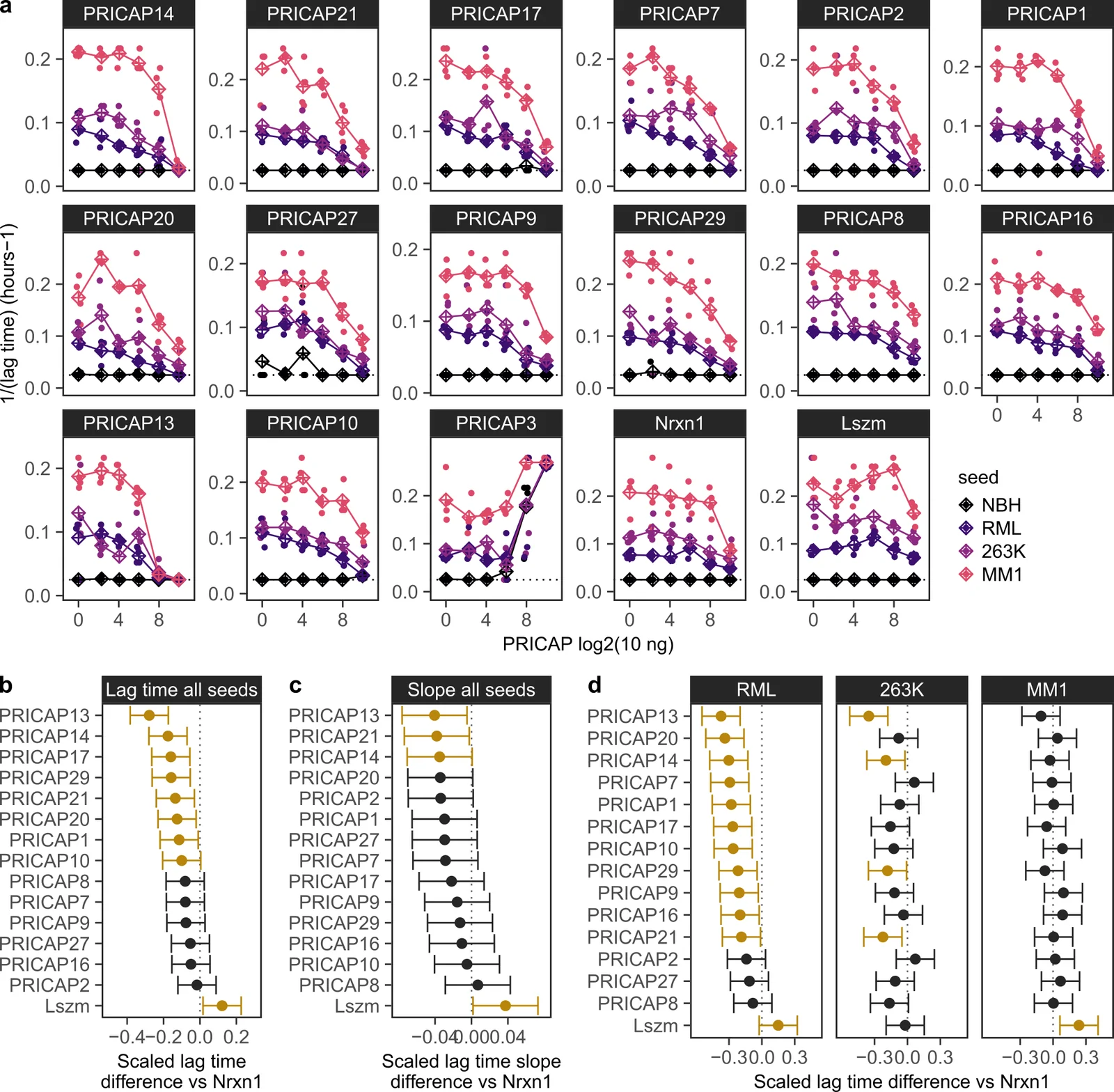
PRICAPs inhibit prion-seeding activity in RT-QuIC assays. RT-QuIC reactions using full-length hamster PrP (23–231) were seeded with 5 ng of brain homogenate protein from mock-infected CD1 mice (non-infectious brain homogenate, NBH), RML-infected CD1 mice, 263K-infected Syrian golden hamsters, and a human case of sCJD-MM1. Reaction mixtures were spiked with PRICAPs in a 4-fold dilution series from 10 µg to 39.1 ng protein per reaction (five concentrations plus a no-PRICAP control). (**a**) ThT fluorescence curves show prion seeding kinetics across PRICAP-seed combinations. (**b–d**) Inverse lag time measurements indicate time to reach fluorescence threshold. Lag times were scaled to the no-PRICAP control and fit to generalized linear models with PRICAP identity, seed, and log dilution as covariates. (**b**) Average lag-time differences across seeds compared to the Nrxn1 control. (**c**) Average lag-time slope differences across seeds compared to Nrxn1. (**d**) Per-seed lag time differences compared to Nrxn1. (**b–d**) show mean lag time differences with 95% confidence intervals. Lszm, lysozyme.

As PRICAPs bind fibril ends, they should impede prion replication by sequestering seeding-competent surfaces. We therefore examined whether PRICAPs interfere with prion-seeding activity using RT-QuIC, a sensitive seed-amplification method widely used in prion diagnostics (28). RT-QuIC monitors and quantifies PrP amyloid formation triggered by exogenous prion seeds using ThT, an amyloid-sensitive dye. Reactions were seeded with Mock, RML, 263K, and sCJD-MM1 brain homogenates and spiked with PRICAPs at dilutions ranging from 10 µg to 39.1 ng per reaction (Fig. 6a; Fig. S10). Controls included Nrxn1 and lysozyme-spiked reactions.

Fifteen of 30 candidate PRICAPs were purified in sufficient quantity to test using RT-QuIC. Encouragingly, 14 of these exhibited anti-prion-seeding activity, as evidenced by dose-dependent extensions of lag times. In many cases, reactions spiked with 10 µg PRICAP showed complete inhibition with no detectable seeding activity. PRICAP3 was a notable outlier, promoting ThT fluorescent products rather than inhibiting seeding, and was therefore not considered further. Nrxn1 did exhibit evidence of inhibition in RT-QuIC assays, perhaps owing to its reported ability to bind fibrils (24, 25), but to a lesser extent than most PRICAPs, while lysozyme had no effect.

To rank PRICAPs by prion seeding inhibition, lag time measurements were fit to a generalized linear model and compared to Nrxn1 to estimate scaled lag time differences (Fig. 6b, c, and d). This verified that PRICAPs exhibited stronger inhibition of prion seeding than Nrxn1. Most PRICAPs showed the strongest inhibition against RML, consistent with their design against structures derived from mouse-adapted scrapie strains (RML or ME7). Overall, PRICAP13, PRICAP14, PRICAP21, PRICAP29, and PRICAP17 exhibited the strongest inhibition of prion seeding.

### PRICAPs delay prion propagation in organotypic cerebellar slice cultures

**Fig 7:**
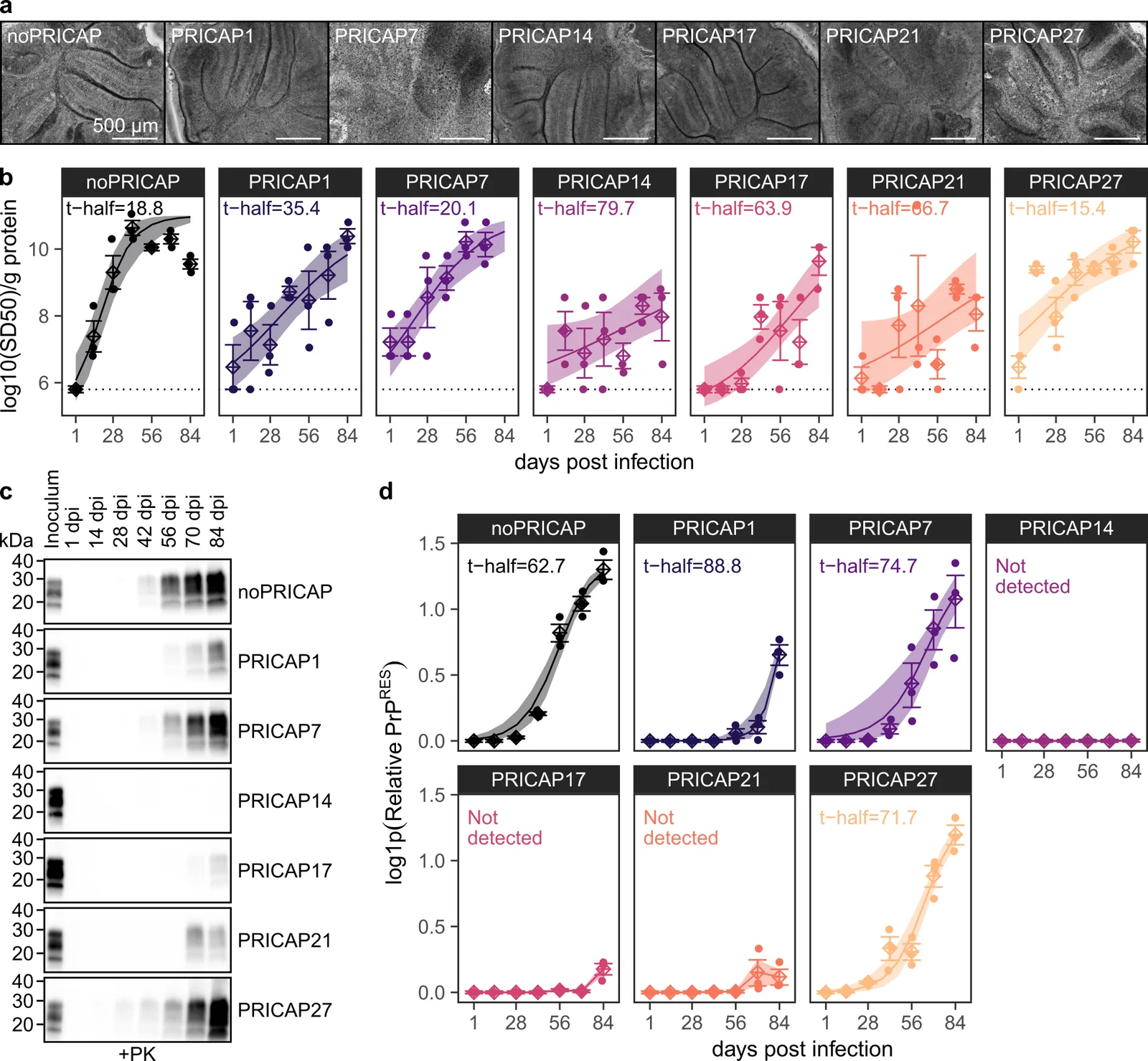
PRICAPs attenuate prion replication kinetics in organotypic cerebellar slice cultures. Wild-type (CD1) cerebellar slices were inoculated with RML scrapie brain homogenates and cultured for up to 84 days (*n = 3* per time point-treatment group). Media were supplemented with or without 100 µg/mL of PRICAP proteins for the entire duration of the experiment. (**a**) Microscopy images show the preservation of cerebellar morphology at 84 days post-infection across treatment groups. (**b**) Prion-seeding activity (SD50) was measured from RT-QuIC at bi-weekly intervals and fitted to two-parameter models to determine the time to reach half-maximal seeding activity (t-half). (**c**) PrP^RES^ was measured in proteinase K-digested slice culture lysates through western blotting with the SAF83 antibody. (**d**) PrP^RES^ measurements were normalized to inoculum signal and fitted to two-parameter models (where possible) to determine the time to reach half-maximal PrP^RES^ signal (t-half) across conditions. (**b** and **d**) The figure panels show model fits and per-time point mean ± SE.

To evaluate whether PRICAPs can engage and interfere with infectious brain-derived prions, we assessed their effect on prion replication in CD1 cerebellar slice cultures infected with RML scrapie. This *ex vivo* paradigm reproduces prion replication kinetics observed in mouse bioassays (29), providing a rigorous model for testing anti-prion activity. Six PRICAPs (PRICAP1, PRICAP7, PRICAP14, PRICAP17, PRICAP21, and PRICAP27) were chosen to test in the slice culture model based on their inhibition of prion seeding activity and stability. Slice culture medium was supplemented with 100 µg/mL of PRICAP for the duration of the experiment, and untreated slices served as controls. Cultures were inoculated with RML prions on day 1 and maintained for 84 days, with lysates collected every 14 days beginning at day 1 post-infection. Cerebellar morphology was comparable across the treatment groups (Fig. 7a). Prion replication was tracked by measuring prion seeding activity (Fig. 7b) and proteinase-K-resistant PrP (Fig. 7c and d).

To rank PRICAPs by the attenuation of prion propagation, both seeding activities and PrP^RES^ measurements were fit to separate two-parameter sigmoidal logistic growth models (Fig. 7b and d) to determine the time to reach half-maximal signal (*t*_1/2_) across groups. Compared to untreated control slices, prion replication kinetics were attenuated in slices treated with PRICAP14, PRICAP21, and PRICAP17, as evidenced by an ~3- to 4-fold extension of seeding *t*_1/2_ and minimal detection of PrP^RES^. Prion attenuation was also seen to a lesser extent in PRICAP1-treated slices, whereas replication in slices treated with PRICAP7 and PRICAP27 was comparable to the controls.

## DISCUSSION

Prion amyloids are widely assumed to replicate through an end-elongation model in which fibril termini act as surfaces that recruit and convert PrP monomers into the misfolded conformer. Direct investigations of this hypothesis have been limited by the lack of molecular probes that selectively recognize disease-associated amyloid surfaces.

Here, we used RFdiffusion and prion cryoEM structures to design a class of fibril-capping proteins, which we term PRICAPs, that interface with prion β-hairpins at fibril termini. From 159 in silico-validated PRICAPs, we selected 30 representatives and verified their binding mechanism, solution stoichiometry, and secondary structure using dot blot assays, TEM, and combined biophysical methods. PRICAPs impeded prion-seeding assays and slowed prion replication in cerebellar slice cultures. Together, these findings support the feasibility of structure-guided targeting of amyloid fibrils, validate that existing prion cryoEM structures can be used for binder design, and demonstrate that fibril termini contribute to infectivity.

PRICAPs have the potential to be developed as prion detection or enrichment reagents, since they preferentially bound recombinant prion fibrils over monomeric PrP and engaged fibril ends. Several pre-analytical prion enrichment approaches have already reportedly improved detection in serum or plasma using diagnostic seed amplification assays, like RT-QuIC (30–35). Therefore, incorporating PRICAPs into similar enrichment workflows, or using them as capture reagents in ELISA assays or for *in situ* labeling of fibril ends, could provide new tools to characterize prion diseases or track disease progression.

Our findings of PRICAP-mediated impairment of prion replication in both RT-QuIC and cerebellar slice cultures demonstrate that amyloid fibril termini contribute to infectivity and are viable therapeutic targets. Promisingly, PRICAPs exhibited evidence of binding to multiple prion strains in both dot blot assays and RT-QuIC, likely owing to their targeting of the β-hairpin conformation in C-terminal PIRIBS lobes that appears relatively conserved across solved prion structures so far (3–8). However, these PRICAPs are not yet at a stage to implement as therapeutics, as the use of rodent-adapted prion structures for PRICAP design might limit their effectiveness against human prions. Furthermore, the possible emergence of “drug-resistant” prions following prolonged treatment with PRICAPs should be assessed *in vivo*, since this was described for small molecules that impeded PrP^Sc^ accumulation (36–38). Strategies for enhancing PRICAP-mediated attenuation of prion replication should also be explored, for example, by incorporating modalities that target prion fibrils for degradation or clearance (39, 40). Nevertheless, while current therapeutic strategies under investigation aim to reduce PrP^C^ expression levels or target PrP^C^ to prevent misfolding (41–49), our findings lend credence to the concept that PrP^Sc^ is also a viable drug target.

Our observation that PRICAPs impaired prion infection in organotypic slice cultures also validates the biological relevance of the existing prion PIRIBS structures. These structures are limited by their origin from fibrils isolated *ex vivo*, often through proteinase K digestion and sodium phosphotungstic acid precipitation (3–8). While infectivity was retained, these treatments raised the question of how representative the fibril preparations were of *in vivo* prion ensembles. Here, we used the cryoEM structure of RML scrapie to design binders that impaired prion replication in cerebellar slices, suggesting that this structure did exist in the inoculum used and is replication-competent.

Prion fibril termini exhibit polarity, and it remains unclear whether terminal surfaces at one or both poles are replication-competent. Here, one pole was arbitrarily targeted by most of the PRICAPs investigated. Encouragingly, the time to reach half-maximal seeding activity was extended by >4-fold in RML-infected cerebellar slice cultures treated with PRICAP14. This suggests that targeting one pole disproportionately affects replication kinetics, perhaps hinting that only one pole is replication competent. However, answering this question requires treating slice cultures with PRICAPs that target the opposite pole of RML, which should be investigated further.

These findings have several important limitations that merit discussion. We relied on non-infectious *in vitro* PrP fibril preparations to characterize PRICAP-PrP binding, which do not fully represent bona fide prion fibrils in brain tissues. Therefore, while PRICAP engagement with their intended target of *ex vivo* prion fibrils is implied by the anti-prion activities of PRICAPs in seeding assays and cerebellar slice cultures, this has yet to be established directly. However, binding experiments with infectious prion preparations must be performed under prion biocontainment conditions, which was not feasible for our study. Additionally, gold nanoparticle-labeling experiments did not conclusively rule out the possibility of PRICAP binding at fibril sides, and hence, developing orthogonal approaches to assess PRICAP localization along the length of a fibril would be valuable. Finally, as PRICAP-PrP binding appears to be sequence-independent, the specificity of PRICAP binding could be more firmly established by examining interactions with non-PrP amyloid fibrils.

In summary, we present PRICAPs as a new tool for mechanistic dissections of prion replication. They provide evidence that prion fibril termini are replication-competent surfaces, which facilitate seeding activity and infectivity, and can be targeted to prevent prion replication.

## MATERIALS AND METHODS

### Computational design of prion-binding proteins with RFdiffusion

RFdiffusion was used to design binding proteins that cap prion fibril ends. Target structures included RML (PDB ID 7TD6), ME7 (PDB ID 8A00), 22L (PDB ID 8EFU), 263K (PDB ID 7LNA), and CWD (PDB ID 9DMZ) fibrils, each truncated into separate N-terminal and C-terminal lobes containing three PrP rungs. For each target, 1,500–4,000 designs were generated using RFdiffusion’s “Complex_beta” model.

To promote end-capping interactions, we employed structure inpainting, which preserves backbone geometry while overwriting the amino acid sequence. A short β-strand segment from the top or bottom prion rung was selected for inpainting and flanked with 25–75 residues of newly diffused structure on both sides. This positioned the designed protein to bind the adjacent prion rung via backbone interactions. Hydrophobic residues near the inpainted region on the middle rung were selected as binding hotspots. Inpainted and hotspot residues are annotated in Fig. S1.

Sequences were generated for each designed structure using ProteinMPNN, with one sequence per structure and a single fastrelax cycle. Binding was validated using AlphaFold2 initial guess, and designs were ranked based on AlphaFold2 metrics. These metrics include predicted aligned area (PAE) for assessing the overall quality of the predicted complexes, predicted local distance difference test (pLDDT) that estimates per-residue confidence, and root mean square deviation (RMSD) of aligned RFdiffusion and AlphaFold2 structures. Proteins with pae_interaction <11.4 were considered for *in vitro* characterization, allowing the inclusion of one binder targeting the CWD N-terminal lobe. For targets with higher binder yields, more stringent filters were applied based on recommendations by RFdiffusion’s developers (19): pae_interaction < 10, plddt_total > 80, and binder_aligned_rmsd < 2. Coordinates for PRICAPs 1, 7, 14, 17, 21, and 27 have been deposited into the ModelArchive repository (https://modelarchive.org/).

### Expression and purification of PRICAPs

RFdiffusion constructs were cloned into pET41a expression vectors with a C-terminal TwinStrep tag for protein purification. Vectors were transformed into BL21 (DE3) star chemically competent cells (Thermo Fisher Scientific) following the manufacturer’s instructions and plated onto LB agar with 50 µg/mL kanamycin for colony selection. Starter cultures grew overnight at 37°C in LB broth with 50 µg/mL kanamycin, diluted 1:50 into fresh LB broth with 100 µg/mL kanamycin and autoinduction reagents, and then incubated at 37°C for 2 h, followed by 24 h at 16°C. The cells were harvested by centrifuging at 5,000 *× g* for 10 min and stored at −80°C.

Frozen cell pellets were thawed and resuspended in BugBuster Master Mix (Millipore Sigma) supplemented with 1× Pierce protease inhibitor tablets at a rate of 5 mL per gram wet cell pellet. After homogenizing, lysates were incubated while rotating at room temperature for 20 min and then centrifuged at 14,000 *× g* for 20 min to remove insoluble material.

Cleared lysates were applied to StreptactinXT 4 Flow resin. Columns were washed with five column volumes of high-salt wash buffer (100 mM Tris and 500 mM NaCl, pH 7.5) followed by 10 column volumes of low-salt wash buffer (100 mM Tris and 150 mM NaCl, pH 7.5). Proteins were eluted in 10 column volumes of elution buffer (100 mM Tris, 150 mM NaCl, and 50 mM biotin, pH 7.5).

Eluted proteins were concentrated to 500 µL using 10,000 MWCO spin filters, cleared by centrifuging at 14,000 *× g* for 5 min, and subjected to size exclusion chromatography on a Superdex 200 Increase 10/300 GL column using 50 mM Tris, 150 mM NaCl, pH 7.5, as running buffer. Protein peaks were collected, quantified via NanoDrop 2000, and stored at −80°C. For slice culture experiments, purified PRICAPs were passed through 3 mL of endotoxin removal resin, sterilized using a 0.2-µm filter, supplemented with 10% glycerol, and stored at −80°C.

### Production of monomeric PrP and PrP fibrils

Recombinant PrP was purified from *E. coli* as described previously (28), using sequences from human (huPrP), hamster (hamPrP), and white-tailed deer (wtdPrP). Briefly, PrP was overexpressed in BL21 cells at 30°C for 30 h using auto-induction. The cells were lysed in BugBuster master mix, and inclusion bodies were isolated via sequential washing and centrifuging in 0.1× BugBuster. Washed inclusion body pellets were then solubilized in 8 M Gdn-HCl, 100 mM sodium phosphate, 10 mM Tris, pH 8.0, and bound to Ni-NTA resin. Proteins were refolded overnight via gradient removal of GdnHCl into 100 mM sodium phosphate and 10 mM Tris, pH 8.0. Protein was eluted in 500 mM imidazole, 100 mM sodium phosphate, and 10 mM Tris, pH 5.8, and dialyzed into 10 mM sodium phosphate, pH 5.8. This monomeric form of PrP was used as substrate for RT-QuIC reactions and for forming recombinant PrP fibrils.

Fibrils were made by shaking monomeric PrP under partial denaturing conditions. Monomeric PrP was concentrated to 12 mg/mL and mixed 1:3 with 8 M guanidine HCl to produce denatured stocks (3 mg/mL PrP, 6 M guanidine HCl) that were stored at −80°C until use. For fibril formation, denatured PrP was diluted 1:3 in buffer to make mixtures of 1 mg/mL PrP, 2 M guanidine HCl, 20 mM sodium acetate (pH 5.0), 10 mM thiourea, and 1 mM EDTA. Mixtures were filtered through a 0.2-µm membrane, aliquoted into 1.5 mL Eppendorf tubes, and shaken at 1,100 rpm and 37°C for 72–96 h at 1,100 rpm.

Type 1 fibrils were formed without zirconium beads, while type 2 fibrils were generated by adding ~50  µL of 0.5-mm zirconium beads to each tube. After shaking, fibrils were dialyzed at least three times in 20 mM sodium acetate (pH 5.0) over 36 h. Fibril formation was confirmed by thioflavin T fluorescence, confocal microscopy, electron microscopy, and proteinase K digestion.

### Circular dichroism (CD)

CD measurements were obtained using a J-1500 CD Spectrometer (Jasco Inc., Easton, Maryland) and a 0.1-cm quartz cuvette. Spectra were collected on PRICAPs at 25°C in 10 mM phosphate (pH 7.2) and 150 mM NaF at a concentration of 0.1 *A*_280_. Experimental spectra were compared to the top five AlphaFold3 predicted models (50) of the PRICAPs with an additional C-terminal twin-STREP tag using the PDB2CD MD server (51). For comparison, delta epsilon magnitudes of predicted spectra were scaled to match those of experimental spectra.

### Static and dynamic light scattering (SLS/DLS)

Static and dynamic light scattering measurements were performed on a Prometheus Panta (NanoTemper Technologies). All measurements were conducted at 20°C.

For SLS experiments, PRICAP samples were dialyzed into either 50 mM Tris, 150 mM NaCl, pH 7.5, or 20 mM sodium acetate (NaAc), pH 5.0, concentrated to approximately 10 mg/mL, and serially diluted to < 1  mg/mL. Samples were passed through a 0.22-µm spin filter before loading into high-sensitivity glass capillaries. Molecular weights were determined by static light scattering using Debye analysis implemented in R. Scattering intensity data were fit to the nonlinear form of the Debye equation:


$$R_{\theta }=\frac{k∙c}{\left(\frac{1}{M_{w}}+2A_{2}∙c\right)}$$

where *R*_θ_ is the excess Rayleigh ratio, *c* is the protein concentration, *k* is the optical instrument constant, *M_w_* is the molecular weight, and *A*_2_ is the second virial coefficient. Nonlinear least-squares fitting was performed to estimate *M_w_* and *A*_2_ from the concentration dependence of the scattering intensity. For DLS experiments, hydrodynamic radii and polydispersity indices (PDI) were determined using the cumulant analysis method within PR Panta Control software. Refractive index values were taken from NanoTemper’s internal buffer database. PrP fibrils (1 mg/mL in 20 mM NaAc, pH 5.0) were sonicated for 0–300 s prior to data collection.

### Fibril dot blot assays

Dot blots were used to assess the binding of PRICAPs to monomeric and fibrillar PrP. PrP samples were serially diluted 2-fold from 500 ng/µL to 0.031 ng/µL (15 dilutions in total), and 1 µL of each dilution was spotted onto nitrocellulose membranes. The eight PrP samples included monomeric and fibrillar forms of human (humPrP), hamster (hamPrP), and white-tailed deer (wtdPrP) PrP: monomeric hamPrP, hamPrP type 2 fibrils, monomeric humPrP, humPrP type 1 fibrils, humPrP type 2 fibrils, monomeric wtdPrP, wtdPrP type 1 fibrils, and wtdPrP type 2 fibrils.

Membranes were dried for 1 h, blocked with 10% skim milk in TBST for 2 h, and washed 3 × 5 min with TBST. Membranes were then incubated overnight at 4°C with PRICAP’s or Neurexin 1 (Nrxn1) negative control, diluted to 850 nM in blocking buffer. After washing 3 × 5 min with TBST, the membranes were incubated with StreptactinXT-HRP (diluted 1:10,000 in blocking buffer) for 2 h, followed by 3 × 5 min washes with TBST. Chemiluminescence signal was developed with the Pierce Femto kit. Dot blot images are shown in Fig. S8.

Signal intensities were quantified using ImageJ (52), scaled from 0 to 1 for each target, and fit to a four-parameter logistic equation to determine logEC50 values:


$$Signal=\min+\;\frac{\max-\;min}{1+\;10^{\left(log10\left(EC50\right)\;-\;log10\left(ng\;protein\right)\right)\;\times \;hill\;slope}}$$

Fibrillar logEC50 values were calculated from five targets (hamPrP T2, humPrP T1, humPrP T2, wtdPrP T1, and wtdPrP T2), and monomeric logEC50 values from three targets (hamPrP, humPrP, and wtdPrP).

In a second experiment, mouse PrP T1 fibrils were sonicated for 0–300 s in a QSonica Q700MPX sonicator before serially diluting 2-fold from 500 ng/µL to 0.031 ng/µL, spotting onto nitrocellulose membranes and proceeding with the above protocol.

### Negative staining and transmission electron microscopy

Prion protein fibril samples were adsorbed for 1 min on either a glow-discharged carbon-coated formvar film on a 400-mesh copper grid or a glow-discharged ultrathin carbon film on lacey carbon support film on a 400-mesh copper grid (Ted Pella, USA). The samples were then negatively contrasted with 2% methylamine tungstate (Nano-W, Nanoprobes, USA). Specimens were imaged in either a Glacios transmission electron microscope (Thermo Fisher Scientific, USA) operating at 200 kV, or a LVEM25 low voltage electron microscope (Delong Instruments, Czech Republic) operating at 25 kV. Prion protein fibril length measurements were made using the ImageJ software package (52) using the segmented line tool and the analyze/measure function.

### Gold nanoparticle electron microscopy

GNP-EM grids were prepared by incubating 0.2 mg/mL fibrils with 0.3 mg/mL PRICAP constructs in 20 mM acetate (pH 5) buffer for 10 min. In total, 5 μL of the solution was blotted onto 300 mesh formvar carbon grids (Ted Pella, Inc., Redding, CA) and incubated for 1 min. Grids were blotted with filter paper before the addition of 10 μL of 1:100 5 nm gold nanoparticles (Nanocs Inc., New York, NY) in 20 mM acetate (pH 5), 1 M NaCl, and 1 mg/mL BSA. Grids were subsequently washed three times with 20 mM acetate (pH 5) and 1 M NaCl, followed by three washes with MilliQ water. The grids were stained with 5 μL of 1.5% (wt/vol) uranyl acetate in water for 1 min before blotting and drying for at least 30 min. Grids were imaged on a Thermo Fisher Scientific Talos F200C operating at 200 kV equipped with a Falcon 4 camera.

GNPs were identified and manually marked using a custom ImageJ macro, which recorded the coordinates of each nanoparticle by clicking. Fibrils were segmented automatically using a custom U-Net-based convolutional neural network trained on approximately 100 manually labeled images. The resulting binary fibril masks were refined and converted into skeleton representations, from which fibril endpoint and branchpoint nodes were extracted. Each GNP was classified as fibril-associated if its coordinates fell within the segmented fibril mask. Fibril-associated GNPs were further classified as being located at fibril ends or fibril sides based on their distance to the nearest fibril endpoint: GNPs within 16 pixels of any endpoint node were labeled as “end-associated,” whereas all remaining fibril-associated GNPs were labeled as “side-associated.” Per-image GNP counts were analyzed in R using a Poisson regression model of the following form: Count ~GNP_type * PRICAP_treatment + Fibril_type + Total_GNPs_per_image, and treatment effects relative to the noPRICAP control were estimated using marginal means and FDR-corrected using the Holm method.

Initial experiments using human and white-tailed deer PrP fibrils yielded 182 images and 959 classified GNPs. A larger data set using sonicated mouse PrP fibrils produced 1,948 images and 13,033 classified GNPs.

### Brain homogenates

Brain homogenates (10% wt/vol) were prepared in PBS by two 30 s sonication bursts, followed by two 10 min centrifugations at 2,000 × *g*. Brain tissues were from a CD1 mouse infected with Rocky Mountain Laboratory scrapie (RML), a golden Syrian hamster infected with 263K scrapie, a non-infected golden Syrian hamster (Mock), and a human sCJD MM1 case.

### RT-QuIC

A modified version of RT-QuIC was used to measure anti-prion-seeding activity of PRICAPs. Briefly, 10% (wt/vol) brain homogenates were diluted to a final concentration of 5 ng/µL protein in PBS supplemented with 0.02% SDS and 0.4% N2. PRICAPs (or Nrxn1 and lysozyme-negative controls) were concentrated to 10 mg/mL and then diluted in brain homogenates to 1 mg/mL. Five 10× PRICAP concentrations were prepared by 4-fold serial dilution: 1 mg/mL, 250 µg/mL, 62.5 µg/mL, 15.6 µg/mL, and 3.1 µg/mL. Diluted brain homogenate without PRICAP was included as a positive control. Ten microliters of each PRICAP-homogenate dilution were added to 96-well plates in quadruplicate, followed by 90 µL of QuIC master mix. Each reaction contained 1× PBS (pH 7.4) supplemented with 162 mM NaCl, 10 nM ETDA, 10 nM ThT, 0.002% SDS, 0.1 mg/mL monomeric hamPrP substrate, 50 ng brain homogenate, and PRICAP (0.1 mg/mL down to 310 ng/mL), or no PRICAP. Plates were sealed and incubated in OMEGA Fluorostar plate readers at 42°C with double orbital shaking (700 rpm) in 1 min shaking/1 min rest cycles. ThT fluorescence was read every 15 min.

Data were exported into csv format and then analyzed in R using the rtquicR package (https://github.com/jslota/rtquicR). Fluorescence signals were scaled by dividing each trace by the mean baseline signal from the first 2 h. Lag times were defined as the time point at which the slope of ThT fluorescence exceeded 0.2 RFU/h. These values were used to calculate inverse lag phase metrics [(lag phase)⁻¹] for each reaction. Lag times from prion-seeded reactions were scaled by dividing by the mean lag times from no-PRICAP control reactions and fit to a Gaussian generalized linear model of the following form: scaled lag time ~PRICAP + seed + log(dilution). PRICAP3 was excluded from the model because it promoted seeding activity. Lag time differences between PRICAPs and Nrxn1 were estimated using marginal means and FDR-corrected using the Holm method.

For quantifying prion-seeding activity in cerebellar slice culture lysates, we used the RT-QuIC protocol described previously for cell culture homogenates (29, 53). Briefly, the lysates were serially diluted 10-fold such that the final amount carried forward for RT-QuIC ranged from 50 ng down to 500 fg total protein (six dilutions × four replicates per sample). Diluted lysates (2.5 µL) were mixed with master mix (47.5 µL) in 384-well plates and run under the same conditions as above for 50 h. Positive RT-QuIC reactions were defined as those that exceeded 10 standard deviations beyond the baseline signal averaged across all wells at the beginning of the reaction. The Spearman-Karber transformation was applied to each sample dilution series to quantify seeding activity as log10(SD50)/g protein.

### Prion organotypic slice culture assay (POSCA)

Cerebellar organotypic slice cultures were prepared from P9-P11 CD1 mice and inoculated with RML brain homogenate as described previously (29, 54, 55). Briefly, cerebelli were dissected, placed in ice-cold Geys balanced salt solution with 1 mM kynurenic acid (GBSSK), and embedded in 2% low-melting point agarose. Sagittal cerebellar sections (275-µm thickness) were then prepared using a Leica VT1200S vibratome and transferred onto Millicell-CM transwell inserts in six-well plates. Cerebellar sections were maintained in 1 mL of slice culture medium (SCM; MEM Eagle’s medium supplemented with 20% horse serum, 1 mg/L insulin, 5.2 mM NaHCO_3_, 30 mM HEPES, 0.1 mg/mL penicillin, and 0.1 mg/mL streptomycin) at 37°C and 5% CO_2_, with SCM being completely exchanged twice per week.

On day 1 post-culturing, 2 µL of 0.1% RML brain homogenate was directly applied onto each slice, corresponding to a dosage of 1.3  ×  10^5^ SD_50_ units/slice. This dosage was chosen because it was previously established to result in prion replication plateauing by about 80 days post-infection (dpi) in these CD1 cerebellar slice cultures (29). Starting on the day of inoculation, SCM was supplemented with or without 100 µg/mL of PRICAP1, PRICAP7, PRICAP14, PRICAP17, PRICAP21, or PRICAP27 for the entire duration of the experiment. Slices were collected every 14 days, starting on day 1 and ending on day 84 post-infection (*n* = 3 per time point-treatment group). Slice culture lysates were prepared in 50 µL of PBS via 8 × 30 s sonication bursts in a QSonica Q700MPX sonicator with a cup-horn at 90% amplitude, with one freeze-thaw cycle at the halfway point. Pierce BCA assays were used to quantify protein content in slice culture lysates, which were then subjected to RT-QuIC to determine seeding activities (described above) and western blotting to detect proteinase K-resistant PrP (described in “Western blotting” below).

To model prion replication and plateau effects in cerebellar slice cultures, RT-QuIC SD50 and PrP^RES^ measurements were each fit to sigmoidal logistic growth models as described previously (29): $P(t)=\frac{P_{max}}{1\;+\;e^{-k(t\;-\;t_{1/2})}}$, where *P(t*) is the amount of PrP^Sc^ at time *t*, *P*_max_ is the maximum concentration of PrP^Sc^ at the plateau phase, *k* is the exponential rate constant, and *t*_1/2_ is the inflection time point at which PrP^Sc^ reaches half of *P*_max_. Prion replication kinetics were compared across treatment groups using *t*_1/2_ values.

### Western blotting

For the detection of proteinase K-resistant PrP (PrP^RES^), 20 μg of slice culture lysate was digested with 25 μg/mL of proteinase K for 1 h at 37°C. Digestions were terminated by diluting in Laemmli buffer (BioRad) with 10 mM dithiothreitol (DTT) (Millipore-Sigma) and 1× Pierce protease inhibitors (EDTA-free) and boiling for 10 min before electrophoresis, which was run on 8%–16% TGX SDS-PAGE gels (BioRad) and at 200 V. Aliquots from the same batch of RML inoculum were digested and electrophoresed in parallel with slice culture lysates to normalize signal across experiments, and 3.3 µg of proteinase K-digested inoculum was run on each gel. The protein was transferred to low-fluorescence PVDF membranes using the Bio-Rad Transblot system.

Membranes were blocked with 5% skim milk diluted in Tris-buffered saline with 0.1% Tween-20 (TBST) for 1 h at room temperature before incubating with SAF83-mouse-anti-PrP (1:5,000, Cayman Chemical 189765) at 4°C overnight. The membranes were then washed four times for 5 min each and incubated with goat anti-mouse-HRP (1:10,000, DAKO P0447) at room temperature for 1 h. The membranes were washed five times for 5 min each and visualized using the Pierce femto-sensitivity ECL substrate kit (Thermo Fisher). Densitometric quantification of western blot signal intensity was performed with ImageJ and normalized to the corresponding inoculum signal within each gel to produce relative PrP^RES^ measurements.

## Data Availability

The data that support the findings of this study are available from the corresponding author upon reasonable request.

## SUPPLEMENTAL MATERIAL

File S1 mbio.00831-26-s0001.csv Calculated metrics for all PRICAP candidates.

Supplemental figures mbio.00831-26-s0002.pdf Figures S1 to S11.

ASM does not own the copyrights to Supplemental Material that may be linked to, or accessed through, an article. The authors have granted ASM a non-exclusive, world-wide license to publish the Supplemental Material files. Please contact the corresponding author directly for reuse.
