# Supplementary material for: AI-designed prion-capping proteins provide evidence that prion fibril ends are replication-competent surfaces that contribute to prion seeding activity and infectivity
Source: mBio. 2026 Aug 17;17(9):e00831-26. doi: 10.1128/mbio.00831-26 (PMC13556240; doi:10.1128/mbio.00831-26)
Supplement: Supplementary file 1 [file mbio.00831-26-s0002.pdf]

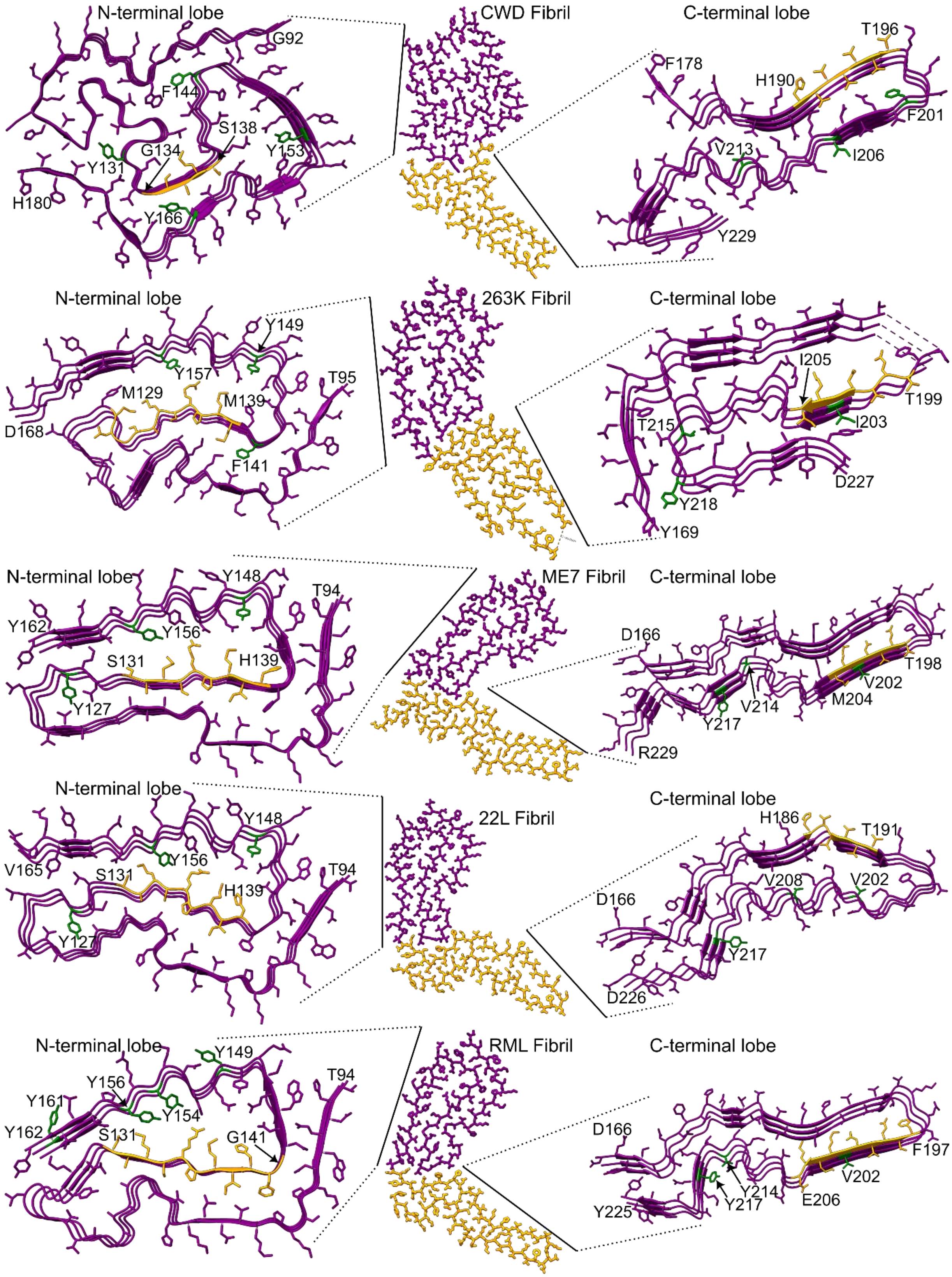
N-terminal lobe
CWD Fibril
C-terminal lobe
G92
F144
S138
Y153
Y131
G134
H180
Y166
F178
T196
H190
F201
V213
I206
Y229
N-terminal lobe
263K Fibril
C-terminal lobe
Y149
Y157
T95
M129
M139
D168
F141
I205
T199
I203
T215
D227
Y218
Y169
N-terminal lobe
Y148
ME7 Fibril
C-terminal lobe
T94
Y162
Y156
S131
H139
Y127
D166
T198
V202
V214
M204
Y217
R229
N-terminal lobe
Y148
22L Fibril
C-terminal lobe
V165
Y156
S131
H139
T94
Y127
H186
T191
V208
V202
D166
Y217
D226
N-terminal lobe
Y149
RML Fibril
C-terminal lobe
T94
Y156
Y161
Y154
Y162
S131
G141
D166
F197
V202
Y214
Y217
E206
Y225

Supplementary Figure 1. Fibril lobe structures used as targets for RFdiffusion inpainting. Shown are fibril structures of RML ME7, 22L, 263K and CWD split into N-terminal and C-terminal lobes, annotated with inpainted regions (yellow) and hotspot residues (green).

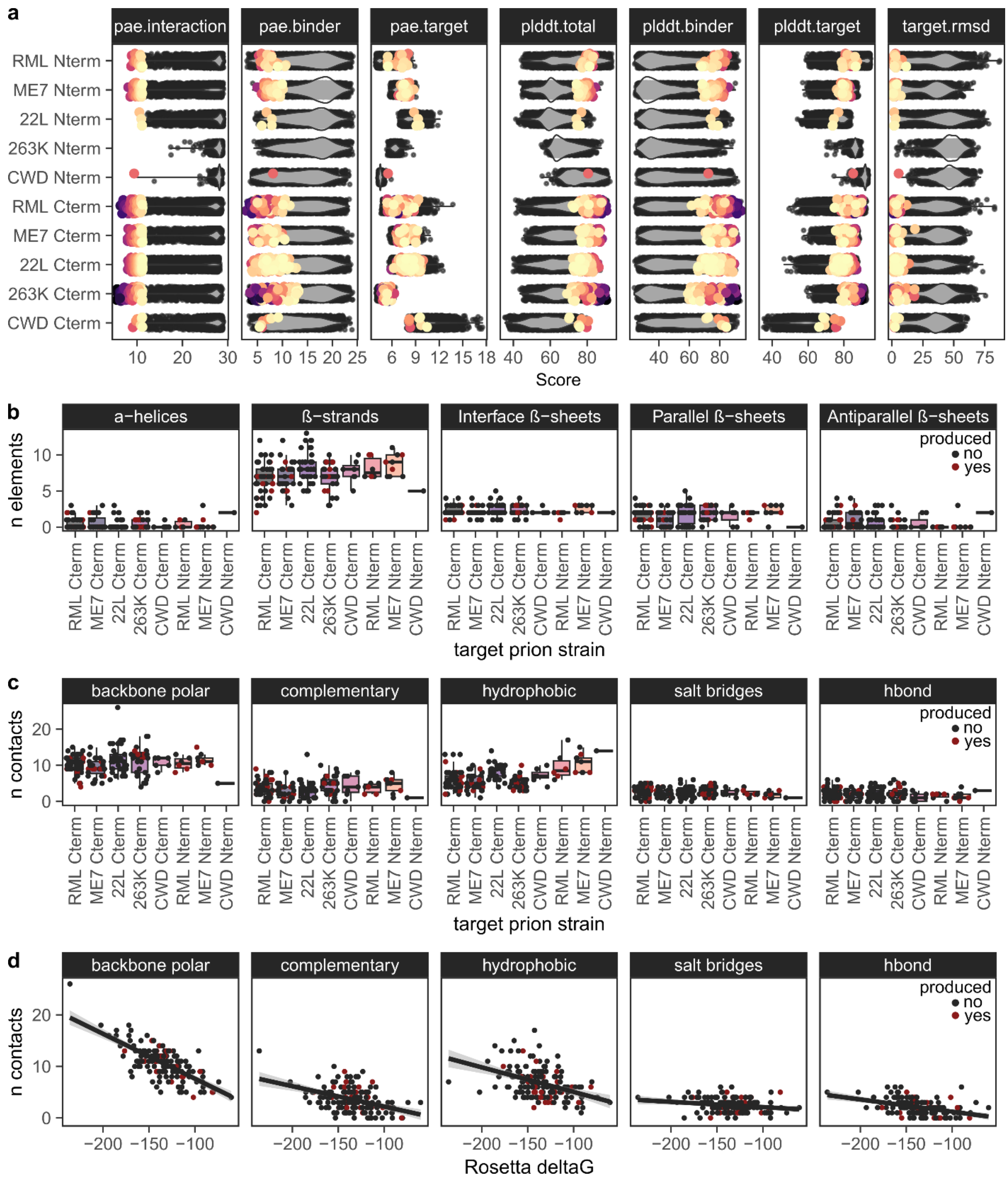

Supplementary Figure 2. Predicted binding metrics of PRICAPs. (a) Complete AlphaFold2 metrics across PRICAPs designed against C- and N-terminal fibril lobe structures of RML, ME7, 22L, 263K and CWD. **(b)** Number of secondary structural elements of PRICAPs, including total alpha-helices and beta-strands, and total, parallel, and antiparallel interfacing beta-sheets between PRICAPs and their target structure. **(c)** Number of residues in each PRICAP that contact their target through backbone polar hydrogen bonding, hydrophobic stacking, salt

bridges, and side-chain hydrogen bonding, as well as complementary residues at PRICAP-PrP interfaces. **(d)** Number of PRICAP-PrP contacting residues against Rosetta deltaG interface scores, stratified by contact type. Boxplots show median (center line), 25th and 75th percentiles (upper and lower hinges), and 1.5*IQR (whiskers).

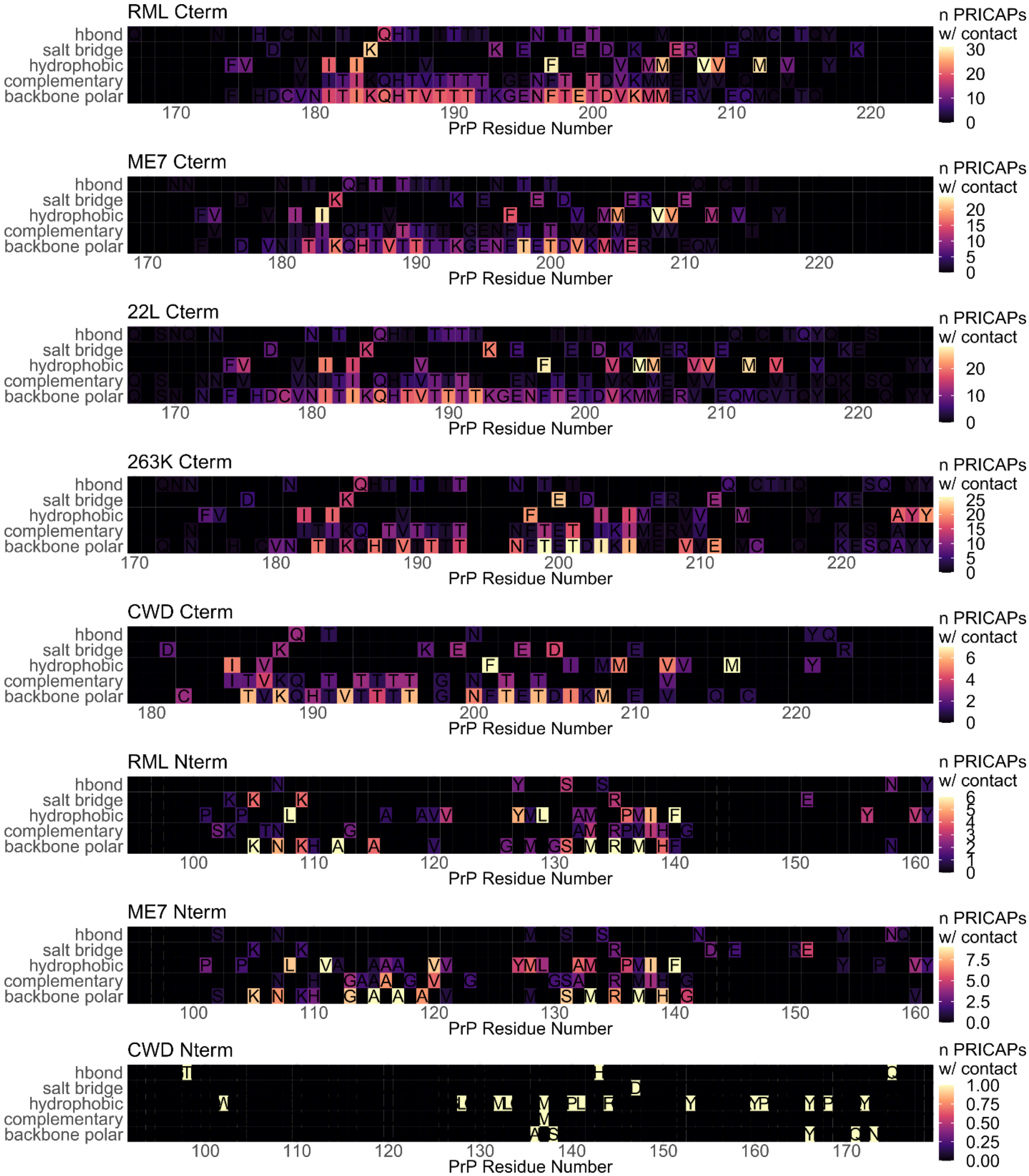

Supplementary Figure 3. PrP residues contacted PRICAPs across prion target structures. Contact maps show number of PRICAPs that contact PrP residues through backbone polar hydrogen bonding, hydrophobic stacking, salt bridges, and side-chain hydrogen bonding, as well as complementary residues at PRICAP-PrP interfaces.

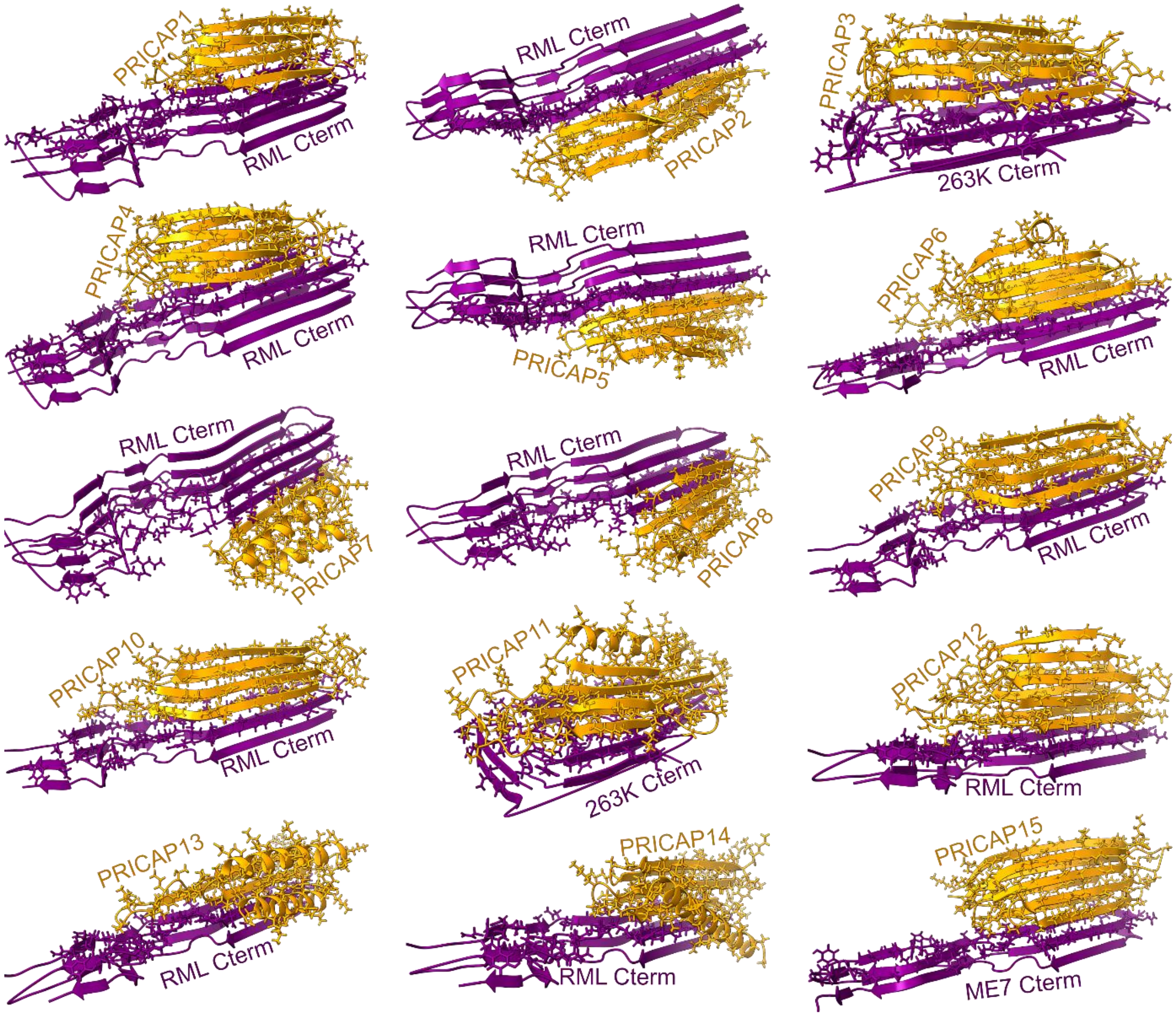

Supplementary Figure 4. Predicted structures of PRICAP1 through PRICAP15 bound to prion fibrils.

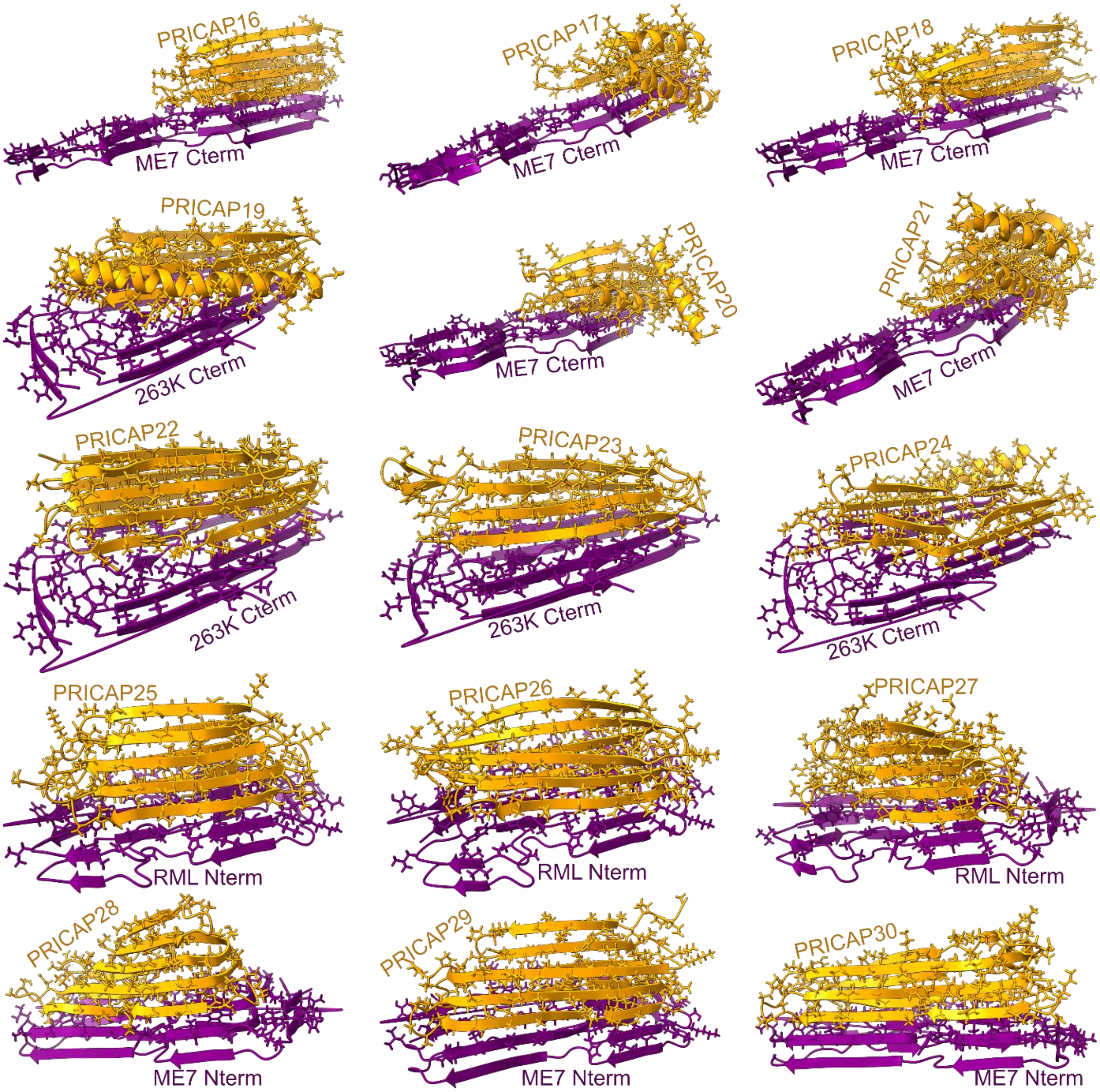

Supplementary Figure 5. Predicted structures of PRICAP16 through PRICAP30 bound to prion fibrils.

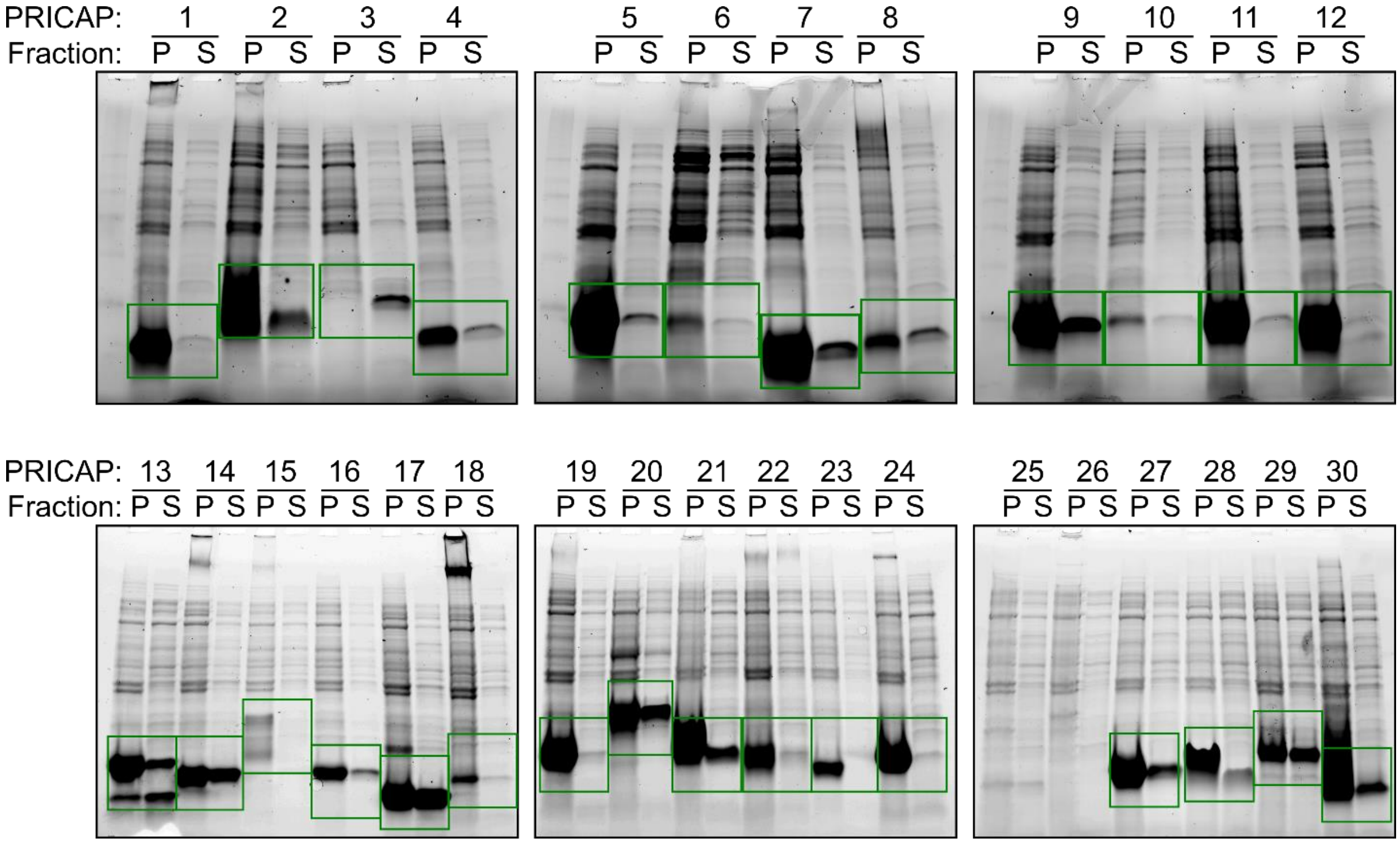

Supplementary Figure 6. Pilot inductions of PRICAPs to screen for expression level and solubility. PRICAPs were expressed in *E. coli* with autoinduction medium for 24 hrs at 28 °C. Cells were then lysed and separated into inclusion body pellets (P) and soluble lysates (S), which were both analyzed via SDS-PAGE using 8-16% gels with stain-free imaging. Bands that presumably correspond to PRICAP proteins are highlighted in a green box.

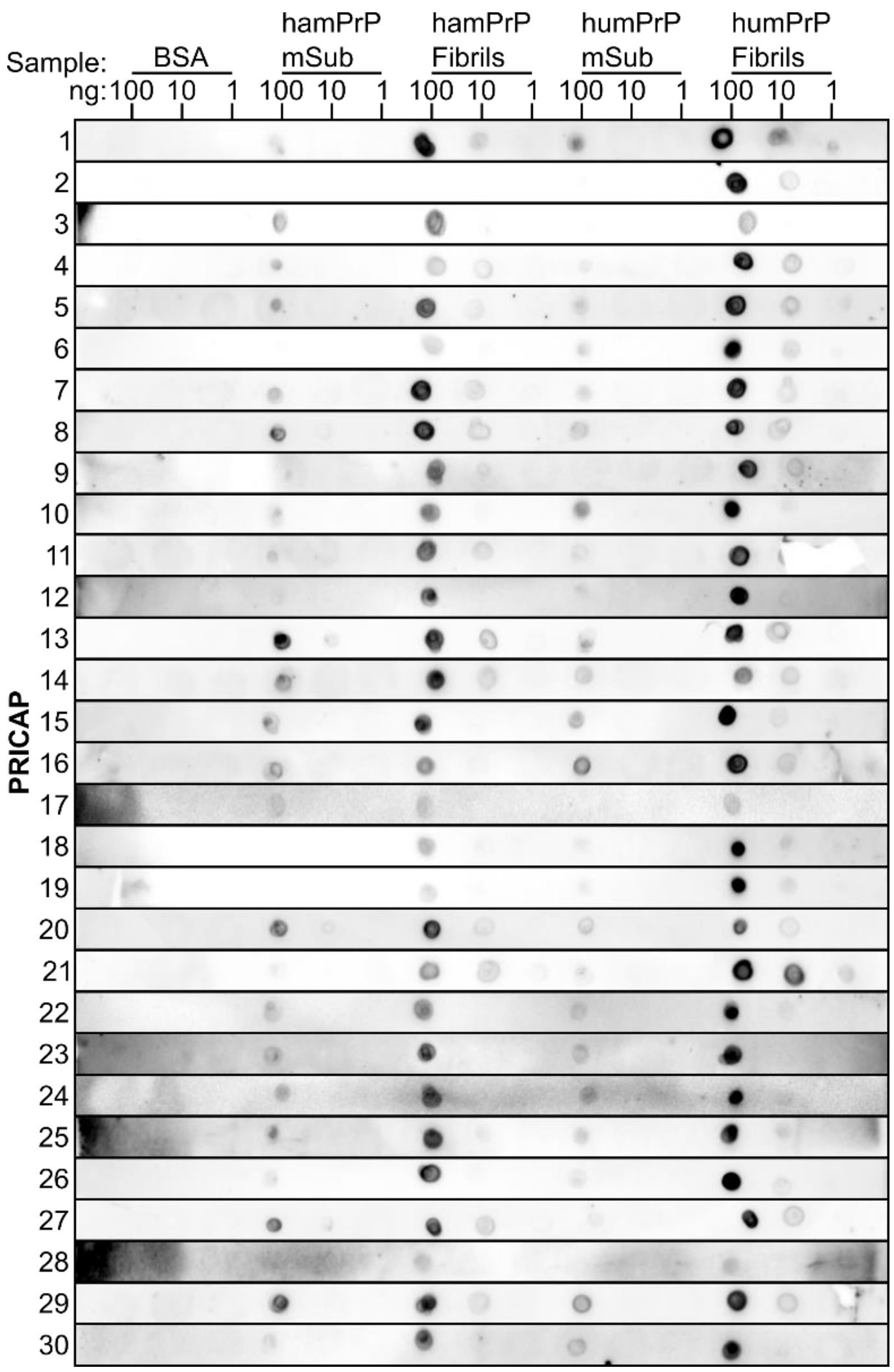

Supplementary Figure 7. Preliminary dot blots to assess PRICAP-fibril binding in *E. coli* lysates. PRICAPs were expressed in *E. coli* with autoinduction medium for 24 hrs at 28 °C and soluble cell lysates were collected. Dot blot assays were then performed by spotting fibrillar and monomeric PrP and BSA (1 – 100 ng) on to nitrocellulose membranes and probing with 20 µL crude PRICAP-containing lysate diluted into 2 mL of blocking buffer.

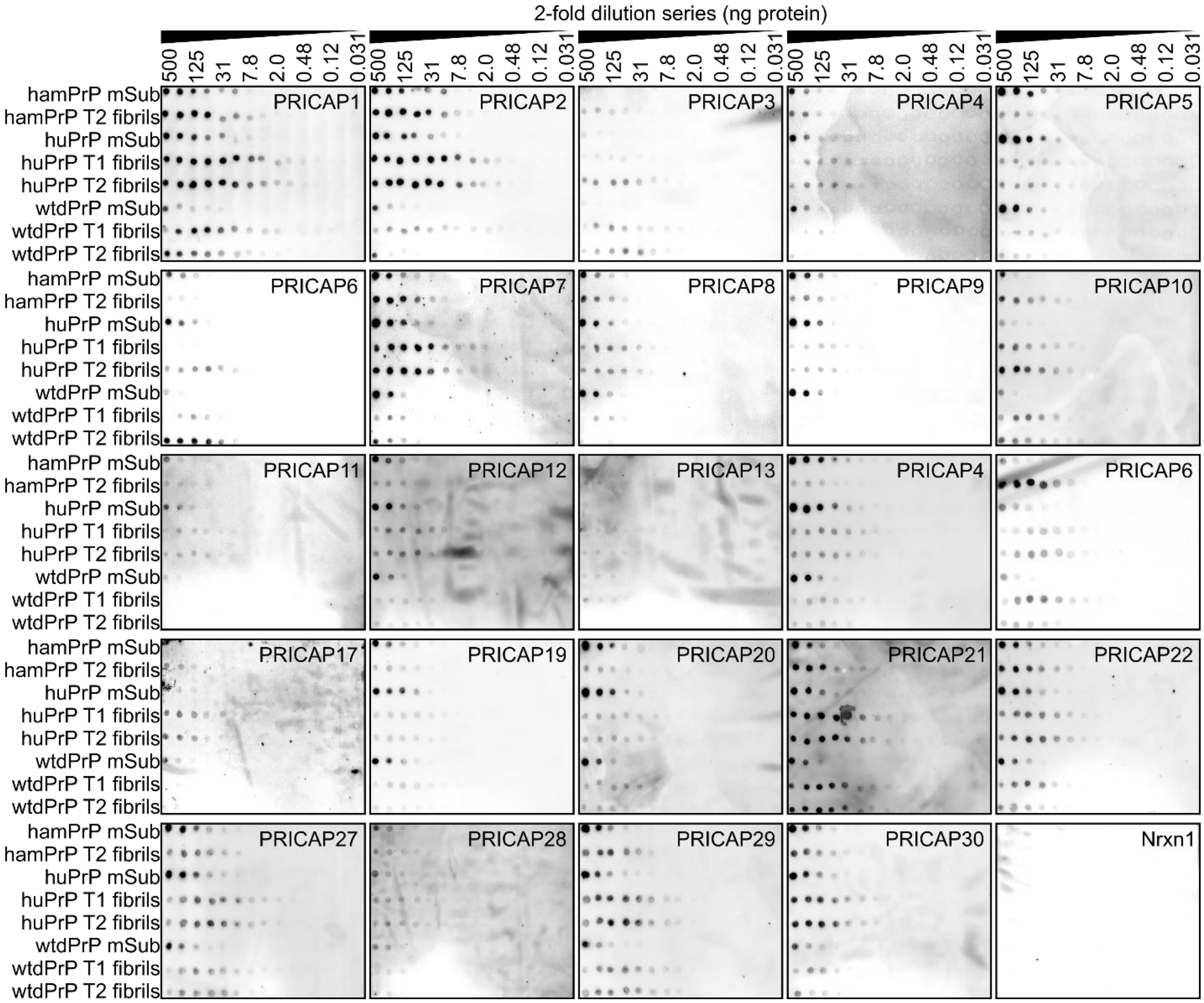

Supplementary Figure 8. Dot blots to assess binding of PRICAPs to monomeric and fibrillar prion proteins (related to Figure 3d). Monomeric (mSub) and Fibrillar (Type 1 and Type 2) forms of PrP from hamster (hamPrP), human (hsaPrP) and white-tailed deer (wtdPrP) were spotted onto nitrocellulose membranes in a 2-fold dilution series from 500 to 0.031 ng (15 dilutions x 8 targets). Membranes were probed with purified PRICAP proteins diluted to 850 nM and binding was detected using StreptacinXT-HRP. Shown are dot blot images, which were quantified using imageJ. Neurexin 1 (Nrxn1) was used as a negative control.

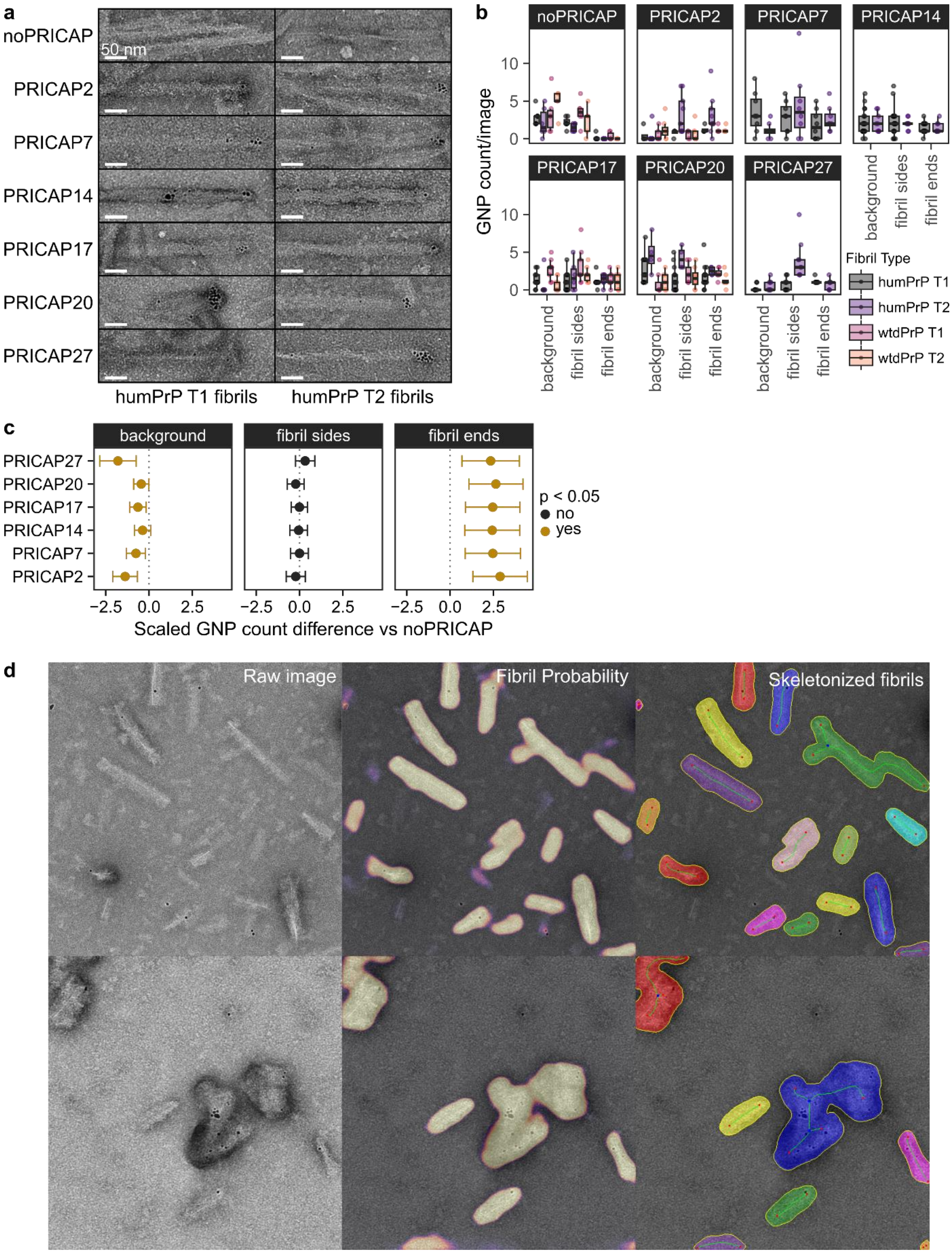

Supplementary Figure 9. Preliminary electron microscopy with gold nanoparticle labelling experiments identifies PRICAPs bound to human recombinant prion fibril ends. (a)

Representative negative stain transmission electron microscopy images of recombinant human prion fibrils (type 1 and type 2) treated with PRICAPs (or no PRICAP) and then stained with streptactinXT-GNP conjugates. **(b)** Raw counts per image of GNPs associated with background, fibril sides, fibril ends, and total GNP multimers. Boxplots show median (center line), 25th and 75th percentiles (upper and lower hinges), and 1.5*IQR (whiskers). **(c)** Distribution of background-, side-, and end-associated GNPs versus no PRICAP control estimated via a generalized linear model. Shown are mean per-image GNP count differences with 95% confidence intervals. **(d)** Representative uncropped TEM images of sonicated mouse PrP fibrils treated with PRICAPs and labelled with GNPs. Images are annotated with probability scores, determined from a UNET image segmentation model, and skeletonized fibrils.

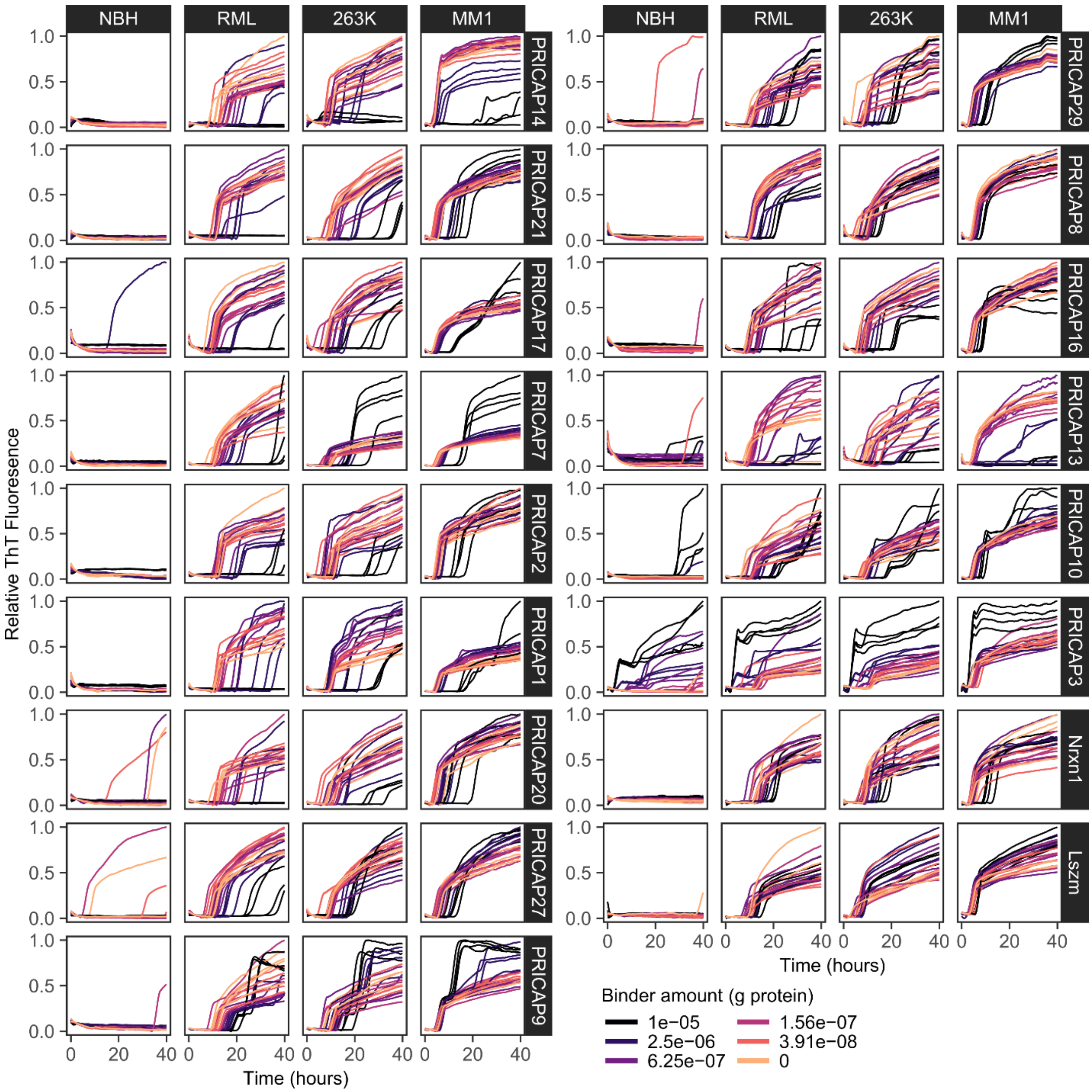

Supplementary Figure 10. RT-QuIC fluorescence traces demonstrating PRICAP-mediated inhibition of prion seeding activity. RT-QuIC reactions using full length hamster PrP (23-231) were seeded with 5 ng of brain homogenate protein from mock-infected CD1 mice, RML-infected CD1 mice, 263K-infected Syrian golden hamsters, and a human case of sCJD-MM1. Reaction mixtures were spiked with PRICAPs in a 4-fold dilution series from 10 µg to 39.1 ng protein per reaction (five concentrations plus a no-PRICAP control). ThT fluorescence curves show prion seeding kinetics across PRICAP-seed combinations.

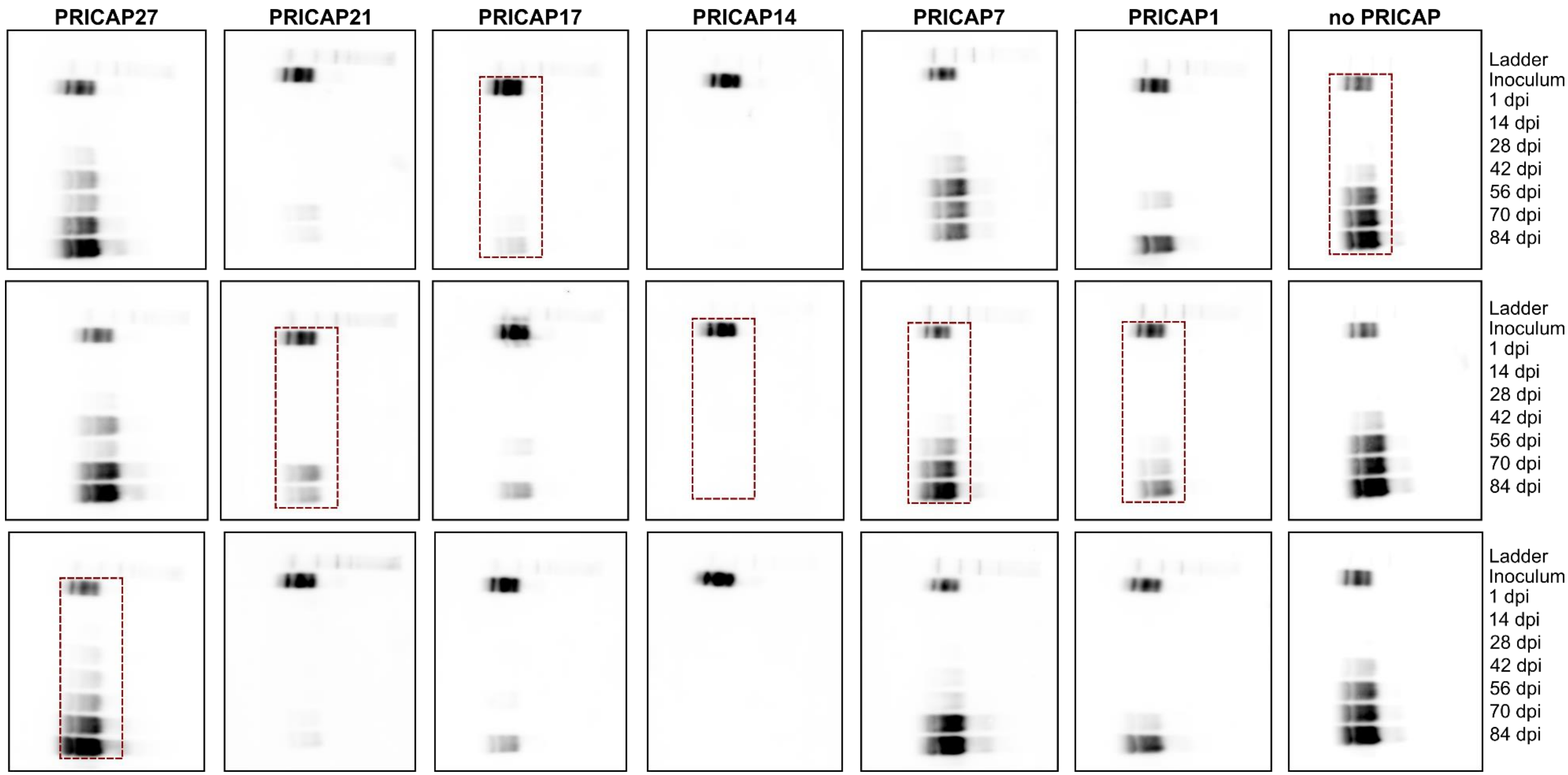
PRICAP27
PRICAP21
PRICAP17
PRICAP14
PRICAP7
PRICAP1
no PRICAP
Ladder
Inoculum
1 dpi
14 dpi
28 dpi
42 dpi
56 dpi
70 dpi
84 dpi
Ladder
Inoculum
1 dpi
14 dpi
28 dpi
42 dpi
56 dpi
70 dpi
84 dpi
Ladder
Inoculum
1 dpi
14 dpi
28 dpi
42 dpi
56 dpi
70 dpi
84 dpi

Supplementary Figure 11. Uncropped western blots images used for PrP^{RES} measurements in cerebellar slice culture lysates. Slice culture lysate (20 µg) was digested with 25 µg/mL of proteinase K in PBS for 1 hr at 37 °C for 1 hr, then western blotted for PrP using the SAF83 antibody. Identical aliquots of RML inoculum (3.3 µg) were included on each gel as a reference point for normalizing PrP^{RES} measurements across gels, which was performed via densitometry with imageJ. Cropped regions shown in Figure 8 are annotated.
